# Ferroptosis-immune crosstalk in CNS diseases: mechanisms and translational insights

**DOI:** 10.3389/fimmu.2026.1807104

**Published:** 2026-04-30

**Authors:** Lili Li, Shuting Wang, Lian Duan, Luyu Zhang, Hongmu Yan, Xiping Chen, Luyang Tao, Yuan Gao

**Affiliations:** 1 Department of Forensic Medicine, School of Basic Medicine, Soochow University, Suzhou, Jiangsu, China; 2 Department of Child and Adolescent Healthcare, Children’s Hospital of Soochow University, Suzhou, Jiangsu, China; 3 Department of Forensic Medicine, The Affiliated Guangji Hospital, Soochow University, Suzhou, Jiangsu, China

**Keywords:** central nervous system disorders, ferroptosis, immune microenvironment, immune response, therapeutic opportunities

## Abstract

Ferroptosis is a form of regulated cell death driven by iron-dependent lipid peroxidation, which plays a pivotal role in regulating the inflammatory-immune microenvironment of central nervous system (CNS) diseases. Mounting evidence indicates that dysregulated iron metabolism and an imbalance in antioxidant defenses can induce ferroptosis in neurons and glial cells while simultaneously remodeling immune cell function, thereby establishing a bidirectional feedback loop that amplifies neuroinflammation and tissue damage. In neurodegenerative diseases, including Alzheimer’s disease (AD), Parkinson’s disease (PD), and amyotrophic lateral sclerosis (ALS), pro-inflammatory cytokines such as TNF-α and IL-1β released by activated microglia upregulate neuronal iron transporters (e.g., DMT1 and TfR1), promoting iron accumulation and ferroptotic cell death. In turn, damage-associated molecular patterns released from ferroptotic cells further potentiate immune activation, forming a self-amplifying cycle. In contrast, within the glioma microenvironment, CD8^+^ T cell-derived IFN-γ suppresses SLC7A11 expression in tumor cells, leading to glutathione depletion and glutathione peroxidase 4 inactivation, thereby triggering ferroptosis and modulating anti-tumor immunity. Although targeting ferroptosis or neuroimmune pathways has shown therapeutic promise in mitigating neurological deficits and enhancing anti-tumor responses, the underlying mechanisms governing ferroptosis-immune crosstalk remain inadequately characterized. Herein, this review systematically summarizes the key biological characteristics of ferroptosis and immune responses, with particular emphasis on their interplay across major CNS disorders (i.e., AD, PD, ALS, multiple sclerosis, stroke, and glioma). Furthermore, we discuss emerging therapeutic strategies encompassing small molecules, immunomodulatory approaches, and nanotechnology-based interventions, highlighting the ferroptosis-immune axis as a promising therapeutic target for CNS diseases.

## 1 Introduction

Regulated cell death is a fundamental biological process essential for tissue homeostasis and organismal integrity, and its dysregulation is increasingly recognized as a central driver of neurological disorders and brain malignancies (1–3). Among the diverse forms of regulated cell death, ferroptosis which is iron-dependent and driven by lipid peroxidation, has emerged as a key contributor to neuropathology. Defined by the accumulation of reactive oxygen species (ROS) and iron-catalyzed lipid peroxides, ferroptosis not only compromises neuronal and glial viability but also actively reshapes the surrounding immune microenvironment (4–6). In turn, immune activation feeds back to exacerbate oxidative stress and ferroptotic vulnerability, establishing a bidirectional amplification loop that accelerates disease progression (7–9).

Mounting evidence implicates ferroptosis in a broad spectrum of central nervous system (CNS) disorders, including acute brain injury, neurodegenerative diseases such as amyotrophic lateral sclerosis (ALS) and Parkinson’s disease (PD), demyelinating disorders such as multiple sclerosis (MS), and primary brain tumors such as glioma (10, 11). In neurodegenerative and demyelinating contexts, disrupted iron homeostasis, excessive ROS production, and lipid peroxidation drive ferroptotic loss of neurons and oligodendrocytes, while simultaneously modulating microglial activation, astrocyte reactivity, and T cell responses (12). Conversely, in tumors, the iron-enriched metabolic landscape and adaptive antioxidant systems render cancer cells particularly susceptible to ferroptosis induction, highlighting a potential therapeutic vulnerability that may synergize with anti-tumor immunity.

Despite these advances, the interplay between ferroptosis and immune regulation in CNS disorders remains insufficiently understood. Several factors contribute to this knowledge gap. First, the cellular heterogeneity of the CNS, encompassing neurons, microglia, astrocytes, oligodendrocytes, and infiltrating immune cells, results in markedly distinct ferroptosis susceptibilities and immune functions. But most studies rely on bulk analyses or simplified *in vitro* systems that fail to resolve cell type-specific dynamics. Second, ferroptosis and immune activation are highly context-dependent processes, varying across disease stages, microenvironmental conditions, and anatomical regions. This feature complicates the interpretation and generalization of experimental findings. Third, the spatiotemporal coordination between ferroptotic signaling and immune responses, including how cytokines, metabolic cues, and lipid mediators modulate ferroptosis sensitivity, remains poorly defined *in vivo*. Finally, while ferroptosis has been extensively studied as an isolated cell death pathway, its integration within broader neuroimmune networks has only recently begun to be explored, leaving critical mechanistic links unresolved.

Addressing these challenges is essential, as the ferroptosis-immune axis represents a convergence point of metabolic, oxidative, and inflammatory signaling. Ferroptosis not only functions as an effector of neuronal and tumor cell death but also regulates immune cell activation, differentiation, and polarization, while immune-derived signals reciprocally shape ferroptosis susceptibility (13, 14). This dynamic crosstalk suggests that ferroptosis is not merely a downstream consequence of pathology but an active driver of disease evolution.

In this review, we provide a comprehensive synthesis of current knowledge on ferroptosis in the CNS, with a particular emphasis on its interactions with immune cells across neurodegenerative diseases, demyelinating disorders, and glioma. We further discuss emerging therapeutic strategies targeting the ferroptosis-immune interface, including small molecules, biologics, nanomaterials, and combinatorial approaches. Finally, we highlight key unresolved questions and propose future directions aimed at enabling precise, context-dependent interventions to mitigate neurodegeneration, promote neural repair, and enhance anti-tumor immunity.

## 2 Biological characteristics of ferroptosis

### 2.1 Iron-dependent accumulation

**Figure 1:**
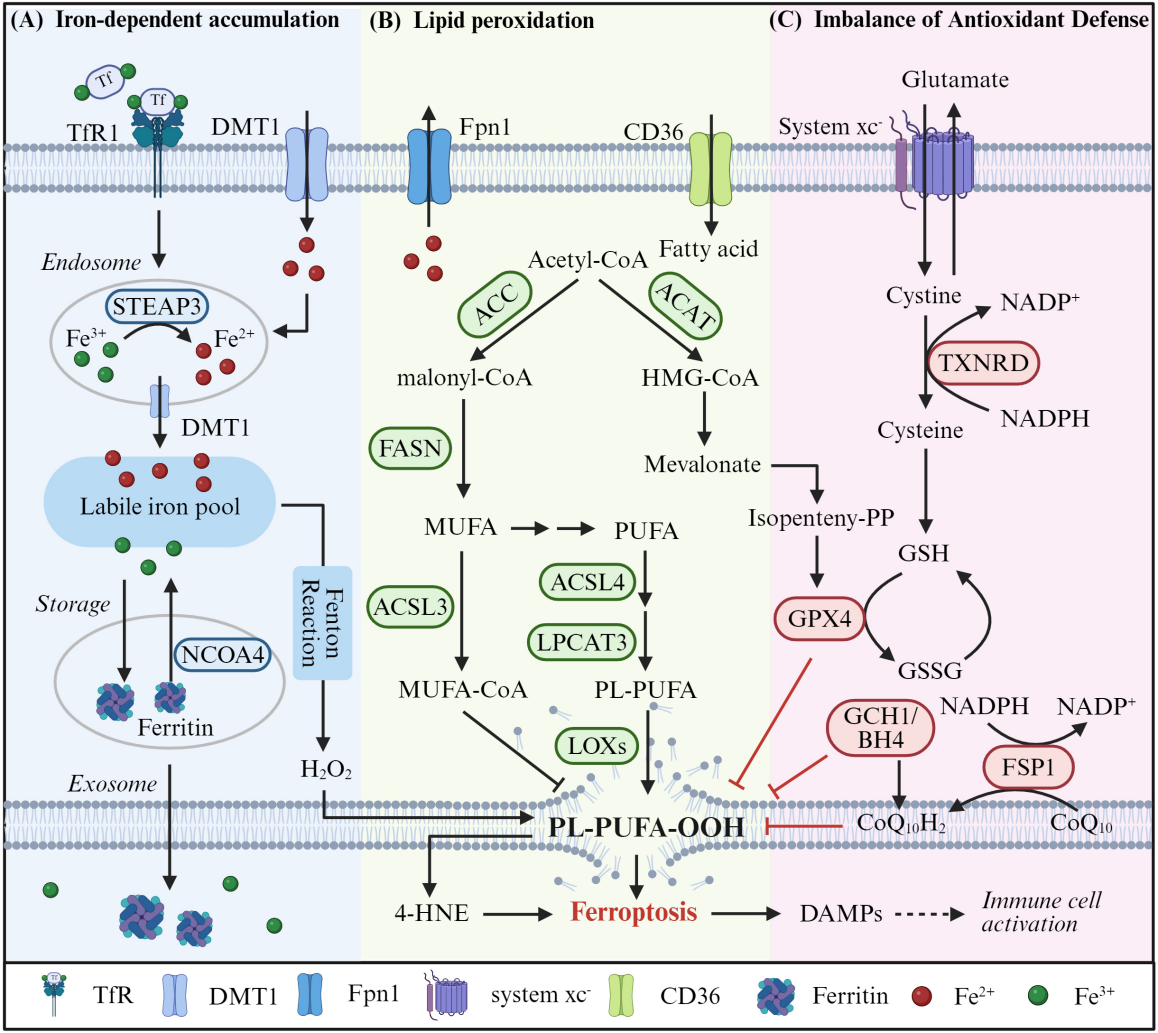
Potential regulatory pathways of ferroptosis. The processes and mechanisms of ferroptosis are intricate and complex, involving the synthesis and accumulation of PUFA-PLs, iron toxicity, and an imbalance between lipid peroxidation and antioxidant defense. **(A)** Excessive intracellular iron uptake (mediated via the TfR1/DMT1 pathway) and the excessive accumulation of iron within the labile iron pool trigger the massive generation of ROS through Fenton reactions. **(B)** Concurrently, lipid metabolic processes generate toxic PL-PUFA-OOH. **(C)** Additionally, once the primary systems counteracting lipid peroxidation, including GPX4, FSP1/CoQH2, and GCH1/BH4-fall out of balance, this triggers the massive accumulation of lipid peroxides on the cell membrane and subsequent membrane rupture, thereby exacerbating ferroptosis.

Cellular iron homeostasis is tightly regulated through coordinated processes of uptake, storage, and export. Circulating Fe³^+^-transferrin complexes bind transferrin receptor 1 (TfR1) to initiate endocytosis, after which Fe³^+^ is reduced to Fe²^+^ by six-transmembrane epithelial antigen of prostate 3 (STEAP3) and transported into the cytosol via divalent metal transporter 1 (DMT1), contributing to the labile iron pool (15). Cytosolic iron is either sequestered in ferritin or exported through ferroportin (FPN), whose activity is negatively regulated by the peptide hormone hepcidin (16). When intracellular iron exceeds buffering capacity, excess Fe²^+^ catalyzes the Fenton reaction, generating highly reactive hydroxyl radicals that drive lipid peroxidation-a central event in ferroptosis (**Figure 1A**). Notably, inflammatory signaling further perturbs iron homeostasis: IL-6 induces hepcidin expression, whereas TNF-α and IL-1β enhance iron uptake via upregulation of TfR1 and DMT1. These coordinated effects promote intracellular iron retention and establish a feedforward loop linking inflammation, iron overload, and ferroptotic vulnerability (10). Despite this framework, the precise molecular mechanisms by which inflammatory cues dynamically regulate iron flux and ferroptosis across different cell types and pathological contexts remain incompletely defined, highlighting an important area for future investigation.

### 2.2 Imbalance between lipid peroxidation and antioxidant systems

Central to the execution of ferroptosis is a disruption in redox homeostasis, wherein oxidative damage overwhelms the cellular antioxidant defenses (17). Neuronal membranes, enriched in polyunsaturated fatty acids (PUFAs), are particularly vulnerable to peroxidation. Free PUFAs are converted to PUFA-CoA by acyl-CoA synthetase long-chain family member 4 (ACSL4) and esterified into phosphatidylethanolamine by lysophosphatidylcholine acyltransferase 3 (LPCAT3), generating PUFA-PE (18). Lipoxygenases, especially ALOX15, directly oxidize PUFA-PE to lipid hydroperoxides (19). This process can also be initiated non-enzymatically by hydroxyl radicals from Fenton chemistry. Accumulation of lipid hydroperoxides disrupts membrane integrity, leading to cell death. In contrast, incorporation of monounsaturated fatty acids (MUFAs) into membrane phospholipids reduces peroxidable double bonds and confers ferroptosis resistance (**Figure 1B**). Neuronal membranes are enriched in PUFAs, explaining their heightened ferroptosis susceptibility, whereas microglia undergo metabolic reprogramming toward MUFA synthesis as a self-protective mechanism.

Balancing this oxidative onslaught is the antioxidant machinery, primarily the GPX4/GSH axis, the FSP1/CoQ10 pathway, and the GCH1/BH4 system (20). GSH serves as a critical intracellular ligand for Fe²^+^, curbing Fenton chemistry and ferroptosis initiation. GPX4 uniquely reduces lipid hydroperoxides, requiring GSH as cofactor. The depletion of GSH precipitates GPX4 inactivation, lipid peroxide accumulation, and ferroptotic cell death. CoQ10 provides a complementary, GPX4-independent antioxidant defense by scavenging free radicals. Its biosynthesis depends on the mevalonate pathway, with FSP1 catalyzing key steps (21). Disruption of this pathway impairs CoQ10 production and predisposes cells to ferroptosis. Similarly, GCH1-driven synthesis of BH4 antagonizes ferroptosis by modulating CoQ10 availability (22) (**Figure 1C**).

Collectively, these insights reveal that ferroptosis in the injured brain results from an imbalance between lipid peroxidation and antioxidant defenses. Therapeutic strategies aimed at reinforcing antioxidant pathways (such as GPX4, CoQ10, and BH4) may offer promising avenues to mitigate ferroptosis-related neurodegeneration.

### 2.3 Immune microenvironment regulation of ferroptosis and its reversibility

The immune microenvironment, including immune cells, inflammatory mediators, and cytokines, plays a pivotal role in modulating neuronal ferroptosis (23). As a form of immunogenic cell death, ferroptosis facilitates the clearance of damaged cells, organelles, and debris via “find me”, “eat me”, and “don’t eat me” signals, thereby maintaining brain homeostasis. The crosstalk between ferroptotic cells and immune components is multifaceted, involving cytokines, transcription factors, and chemokines.

Previous studies have found that activated immune cells regulate ferroptosis through key signaling pathways such as NF-κB, JAK/STAT, and cGAS-STING (24). Under physiological conditions, NF-κB is sequestered in the cytoplasm by IκBα. Upon stimulation by ROS, damage-associated molecular patterns (DAMPs), or lipopolysaccharide (LPS), NF-κB translocates to the nucleus, driving proinflammatory cytokine expression and exacerbating both inflammation and neuronal ferroptosis. Moreover, in a chronic cerebral hypoperfusion mouse model, recent studies reported that dimethyl fumarate (Nrf2 activator) could inhibit NF-κB-mediated inflammation and upregulate NQO1, GPX4, and HMOX1 to protect neurons from oxidative ferroptotic damage (25, 26). Proinflammatory cytokines such as IL-6 and IFN-γ can activate the JAK/STAT1/3/IRF1 axis, repressing mRNA expression of critical Xc^–^ system components SLC3A2 and SLC7A11. This suppression leads to intracellular glutathione depletion, facilitating ferroptosis (27). Additionally, activation of the cGAS-STING pathway has been linked to ferroptosis sensitization. For instance, in human pancreatic cancer cells, erastin-induced ferroptosis upregulates STING, which interacts with mitochondrial fusion proteins MFN1/2 to augment ROS production and lipid peroxidation. Knockdown of STING or MFN1/2 diminishes ferroptosis sensitivity and attenuates erastin’s anti-tumor efficacy (28).

Ferroptotic neurons can emit lipid mediators (such as 5-HETEs, 11-HETEs, and 15-HETEs) that function as “find me” signals, which recruit immune cells to sites of neuronal damage. Concurrently, ferroptotic cells release DAMPs, notably HMGB1, activate NF-κB-dependent inflammatory cascades by binding to toll-like receptors on immune cells. Activation of the HMGB1-AGER axis further stimulates macrophage secretion of proinflammatory cytokines TNF-α and IL-1β (29). Paradoxically, ferroptosis also facilitates immune evasion. Neuronal ferroptosis is associated with upregulation of cyclooxygenase-2 and consequent synthesis of prostaglandin E2 (PGE2). In tumor microenvironment, PGE2 skews T helper cell differentiation toward Th2 and Th17 phenotypes while inhibiting Th1 responses. It also suppresses the anti-tumor activities of natural killer cells, dendritic cells, and cytotoxic T lymphocytes (30, 31).

Together, these findings highlight a bidirectional positive feedback loop wherein neuroinflammation amplifies ferroptosis, and ferroptosis exacerbates neuroinflammation. Therapeutically, disrupting this cycle such as through iron chelation to reduce neuronal iron overload holds promise for mitigating ferroptosis-associated neurodegeneration. Targeting the inflammation-immunity-ferroptosis axis thus emerges as a compelling strategy for neurological disease intervention.

## 3 Immune cells in brain pathophysiology

The activation and functional changes of immune cells are hallmark pathological alterations in neuropathological diseases or injuries, and play a crucial role in the pathophysiology and progression of these conditions in the CNS. The regulatory mechanisms of immune cell activation are complex and diverse, including but not limited to metabolic disorders, oxidative stress, and ferroptosis. Notably, a positive feedback loop may exist between immune cell activation and ferroptosis, and blocking this feedback loop might be might be one of the methods to mitigate neuropathological damage (32). Therefore, targeting ferroptosis, immune cell activation, or the interaction between the two may represent a potential therapeutic strategy for neurological diseases. However, the specific details of this mechanism still require further investigation (**Table 1**).

**Table 1:** Pathophysiological functions of innate and adaptive immune cells.

Cell type	Disease landscape	Pathophysiological functions	Underlying mechanism	References
Microglia	Alzheimer’s disease; Parkinson’s disease; Amyotrophic Lateral Sclerosis; Glaucoma; Huntington’s disease; Psychiatric disorders; Ischemic stroke; Traumatic brain injury	Secrete pro-inflammatory cytokines and chemokines; Clear cellular debris and phagocytose misfolded proteins; Regulate synaptic pruning and remodeling; Induce formation of reactive astrocytes; Display MHC I and II as APCs;	Iron intracellular accumulation promote transformation into pro-inflammatory phenotype and glycolysis phenotype	(50–52)
Astrocyte	Alzheimer’s disease; Parkinson’s disease; Amyotrophic lateral sclerosis; Huntington’s disease; Ischemic stroke; Multiple sclerosis; Traumatic brain injury	Cause neuroexcitotoxicity by regulating glutamate levels; Secrete chemokines; Regulates brain iron concentration; Secrect IL-15 to increase CD8^+^ T cell infiltration and IL-33 to recruit Tregs;	Iron intracellular accumulation trigger neurotoxicty and induce reactive astrocyte proliferation	(53–55)
Neutrophil	Alzheimer’s disease; Ischemic stroke; Spinal cord injury; Multiple sclerosis; Traumatic brain injury	Mediate BBB damage and invasion of the CNS; Secrete NETs and MPO to induce neurotoxicity; Generate ROS to amplify inflammatory response	Iron is involved in the formation of NETs; Intracellular ROS accumulation promotes NETs release	(56, 57)
CD4^+^ T cell	Alzheimer’s disease; Parkinson’s disease; Multiple sclerosis; Uveitis; Glaucoma; Diabetic retinopathy	Th1 cell secretes pro-inflammatory cytokines (i.e., IFN-γ, TNF-α, TNF-β, and IL-1β) to enhance innate immunity	Iron accumulation inhibit Th1, Th2, and Th17 cells differentiation and activity; promote Th1 and Th2 cells immune response as adjuvant	(58)
CD8^+^ T cell	Alzheimer’s disease; Parkinson’s disease; Amyotrophic lateral sclerosis; Susac syndrome	Induce neuronal apoptosis by releasing perforin and granzyme or expressing FasL; Produce pro-inflammatory cytokines (i.e., TNF-α, IFN-γ)	Iron intracellular accumulation promote differentiation of CTL	(59)

### 3.1 Microglia

Microglia are central regulators of neuroinflammation and iron homeostasis in the CNS. Under pathological conditions, they undergo phenotypic polarization toward pro-inflammatory (M1-like) or anti-inflammatory (M2-like) states, a process tightly modulated by oxidative stress and iron availability. Accumulating evidence indicates that inflammatory mediators promote iron accumulation in microglia, which in turn drives M1-like polarization characterized by elevated secretion of pro-inflammatory cytokines and enhanced ROS production (12, 33). This feed-forward loop amplifies oxidative stress and facilitates neuronal ferroptosis.

Notably, despite their pro-inflammatory profile, microglia exhibit a relative resistance to ferroptosis compared with neurons (34). This resistance has been attributed to multiple intrinsic protective mechanisms, including inducible nitric oxide synthase (iNOS)-mediated scavenging of lipid radicals and activation of Nrf2-dependent antioxidant programs, which enhance glutathione synthesis and GPX4 activity (35). However, the relative contribution of these pathways remains incompletely resolved. While some studies emphasize the dominant role of iNOS and Nrf2 signaling, others propose that metabolic reprogramming toward MUFA synthesis constitutes a key adaptive mechanism that limits lipid peroxidation (36–38). The lack of consensus highlights the complexity of ferroptosis regulation in microglia and suggests that distinct protective programs may operate in a context-dependent manner.

Importantly, whether pro-inflammatory microglia themselves become more susceptible to ferroptosis remains controversial. On the one hand, pro-inflammatory activation is associated with increased iron loading and lipid peroxidation, implying heightened ferroptotic vulnerability (12). On the other hand, M1-like polarization has also been reported to reinforce antioxidant defenses, including upregulation of GPX4 and Nrf2, thereby preserving microglial viability under oxidative stress (39). These seemingly contradictory findings may reflect differences in experimental models, disease stages, and microenvironmental conditions.

A key limitation of current studies is the lack of single-cell resolution, which obscures the heterogeneity of microglial subpopulations and their distinct ferroptosis states *in vivo*. Furthermore, most mechanistic insights are derived from simplified *in vitro* systems that fail to capture the dynamic crosstalk between microglia, neurons, and astrocytes. Given that paracrine signaling and metabolic coupling are likely to critically shape ferroptosis susceptibility, future studies integrating single-cell omics and spatially resolved approaches will be essential to delineate the context-dependent roles of microglia in ferroptosis-immune crosstalk.

### 3.2 Neutrophils

Neutrophils rapidly infiltrate the CNS following injury or inflammation, acting as key effector cells in oxidative burst and tissue damage. These innate immune cells not only release large amounts of ROS directly inducing oxidative injury, but also form neutrophil extracellular traps (NETs) enriched with oxidized DNA and histones (40). Through direct membrane contact or indirect signaling with neurons and oligodendrocytes, NETs initiate and exacerbate chain reactions of lipid peroxidation. Specific NET components, such as HMGB1, can activate cell surface Toll-like receptors, thereby upregulating pro-ferroptotic proteins like ACSL4 while suppressing anti-ferroptotic proteins such as GPX4 and FSP1 (41). In models of ischemic stroke, glioma, and neurodegenerative diseases, neutrophil-derived MPO and NETs have been shown to co-localize with ferroptosis-specific markers in neurons and glial cells, including GPX4 downregulation, ACSL4 upregulation, and lipid ROS accumulation (42). Notably, NETs can also activate microglia, amplifying neuroinflammation and creating a vicious cycle that mutually reinforces ferroptosis.

Furthermore, NETs can disrupt the blood-brain barrier (BBB) through the release of matrix metalloproteinases, facilitating peripheral iron ion infiltration into the brain parenchyma and further creating a pro-ferroptotic microenvironment. Zhu Y et al. further revealed that NETs exacerbate BBB damage after TBI by mediating endothelial cell ferroptosis via the ZBP1/FSP1 signaling axis, providing a potential theoretical basis for therapeutic strategies targeting ZBP1 and FSP1 (43, 44).

### 3.3 T lymphocytes

T lymphocytes are central mediators of adaptive immunity, endowed with the capacity to recognize antigenic peptides and orchestrate context-specific immune responses. Broadly, T cells are classified into CD4^+^ helper T cells and CD8^+^ cytotoxic T cells, which coordinate immune activation through cytokine secretion and direct cytolytic activity, respectively (45). Emerging evidence indicates that ferroptosis constitutes a critical metabolic checkpoint in T cell biology, yet its impact is highly subset-specific and context-dependent rather than uniformly protective or detrimental (46).

Recent studies reveal that distinct T cell subsets exhibit markedly different susceptibilities to ferroptosis, thereby shaping immune outcomes across pathological settings. CD8^+^ effector T cells, particularly within the tumor microenvironment, are highly vulnerable to ferroptosis. Mechanistically, upregulation of the scavenger receptor CD36 enhances the uptake of oxidized polyunsaturated fatty acids, leading to excessive accumulation of PUFA-OOH and subsequent ferroptotic cell death, ultimately impairing antitumor immunity (47). In contrast, regulatory T cells (Tregs) display pronounced resistance to ferroptosis, largely due to the activation-induced upregulation of GPX4 and reinforcement of glutathione-dependent antioxidant systems, which enable their persistence in oxidative and immunosuppressive niches (48). This selective resistance provides a competitive survival advantage for Tregs, thereby reinforcing immunosuppression in tumors and chronic inflammatory conditions.

In addition to subset-specific differences, iron metabolism serves as a key determinant of T cell fate and function. In CD4^+^ T cells, intracellular iron availability modulates lineage commitment, where iron sufficiency promotes differentiation toward pro-inflammatory Th1/Th17 phenotypes, whereas iron restriction limits pathogenic T cell responses (49). These findings suggest that ferroptosis is not merely a terminal cell death pathway but also functions as a regulatory node linking metabolic cues to immune cell differentiation. However, several unresolved issues remain. First, conflicting evidence exists regarding the overall ferroptosis sensitivity of T cells, as some studies suggest intrinsic resistance under physiological conditions, whereas others demonstrate heightened vulnerability in disease-specific microenvironments such as tumors or inflamed CNS tissues. This discrepancy likely reflects differences in metabolic states, lipid availability, and microenvironmental stressors. Second, most current insights are derived from *in vitro* systems or tumor models, with limited understanding of T cell ferroptosis dynamics in neuroinflammatory diseases. Finally, the interplay between ferroptosis and T cell exhaustion, a key feature of chronic inflammation and cancer, remains insufficiently defined. Addressing these gaps will be essential for delineating how ferroptosis shapes T cell-mediated immunity across diverse CNS disorders.

## 4 Crosstalk between ferroptosis and immune cells underpinning neurological diseases

**Figure 2:**
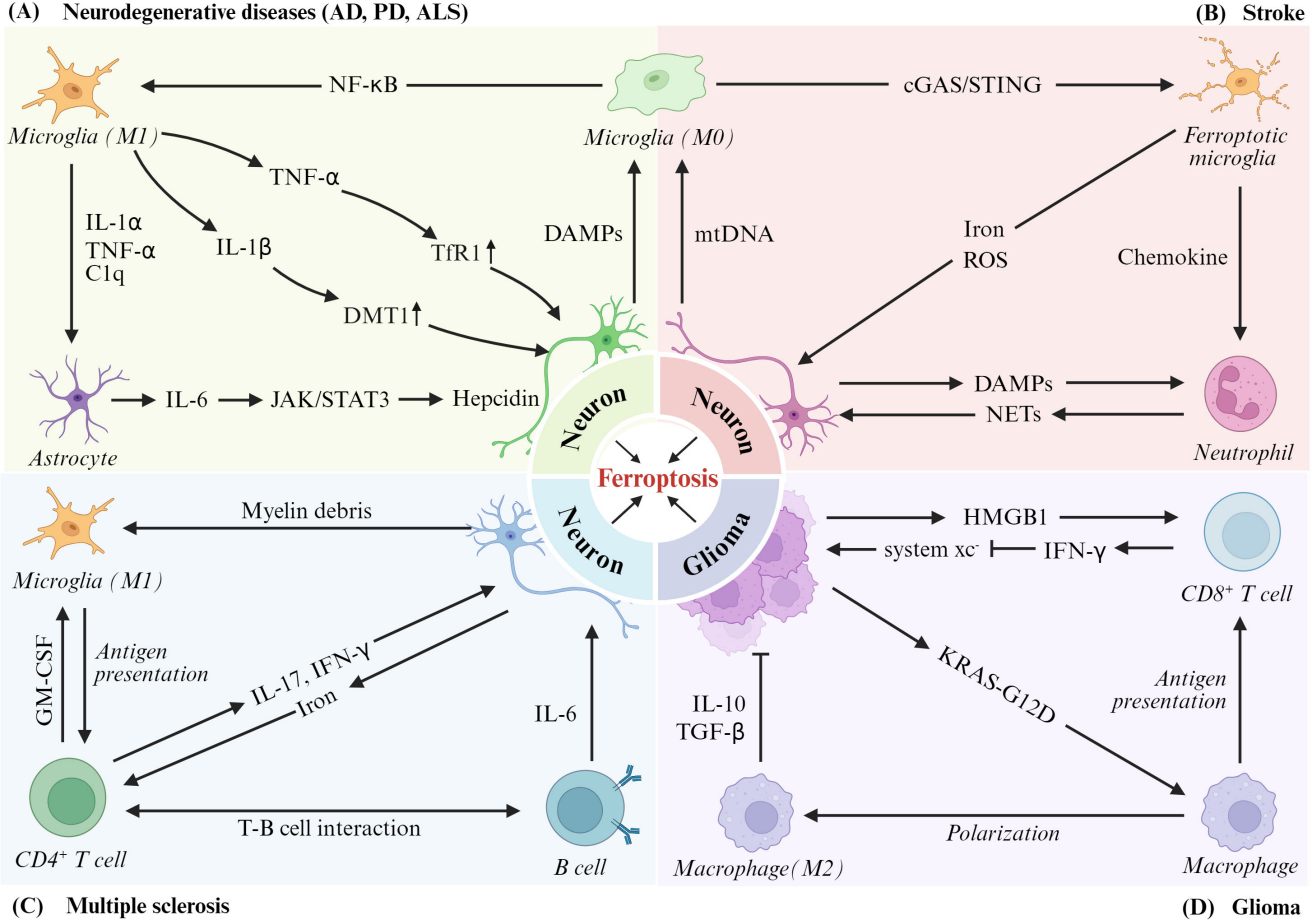
Feedback regulatory loops of ferroptosis-immune response interactions in various central nervous system diseases. **(A)** In neurodegenerative diseases (AD, PD, ALS), NF-κB-activated M1 microglia secrete inflammatory cytokines (TNF-α and IL-1β) to upregulate neuronal iron transporters (TfR1 and DMT1) and trigger ferroptosis. While DAMPs from ferroptotic neurons further amplify microglial activation. Actived by IL-1α and C1q, astrocytes secrete IL-6 to neurons and promote the secretion of hepcidin via JAK/STAT3 signaling, further inducing neuronal ferroptosis. **(B)** In stroke, cGAS/STING-mediated microglial ferroptosis releases iron/ROS and recruits neutrophils, amplifying neuronal injury via DAMPs and NETs. **(C)** In MS, B cells and CD4^+^ T cells secrete pro-inflammatory cytokines to exacerbate neuronal demyelination and iron accumulation, promoting neuronal ferroptosis. **(D)** In glioma, KRAS-G12D-activated M2 macrophages and CD8+ T cells modulate system xc- and IFN-γ signaling to suppress tumor ferroptosis, supporting tumor progression.

Ferroptosis, a regulated form of iron-dependent lipid peroxidation-driven cell death, has emerged as a critical pathological mechanism in diverse neurological disorders, including AD, PD, ALS, MS, stroke, and glioma (60–62). Mechanistically, ferroptosis mediates neurodegeneration by modulating neuronal and glial cell viability, while simultaneously influencing immune cell activation and neuroinflammatory cascades (**Figure 2**). Notably, the role of ferroptosis-immune crosstalk differs fundamentally across these conditions. In neurodegenerative diseases, ferroptosis is predominantly detrimental, driving neuronal loss and amplifying neuroinflammation. In glioma, however, it represents a therapeutic vulnerability, as inducing ferroptosis in tumor cells enhances immunogenic cell death and primes anti-tumor immunity. This fundamental duality underscores the context-dependent nature of ferroptosis-immune crosstalk and informs disease-specific therapeutic strategies. In the following sections, we examine the mechanisms underlying ferroptosis-immune crosstalk in each of these conditions, highlighting both disease-specific features and shared pathogenic principles.

### 4.1 AD

AD is a neurodegenerative disease characterized by memory loss and cognitive decline, with hallmark pathological changes including β-amyloid (Aβ) accumulation and neurofibrillary tangles. The pathogenesis of AD is complex and diverse, including but not limited to immune cell infiltration, neuroinflammation, lipid peroxidation, and ferroptosis (63, 64). Increasing evidence implicates ferroptosis as a key effector pathway interwoven with immune dysregulation, lipid peroxidation, and iron metabolism abnormalities in AD pathogenesis. However, the mechanism of ferroptosis and immune cells themselves and their interactions in the pathological changes of AD remains unclear.

**Figure 3:**
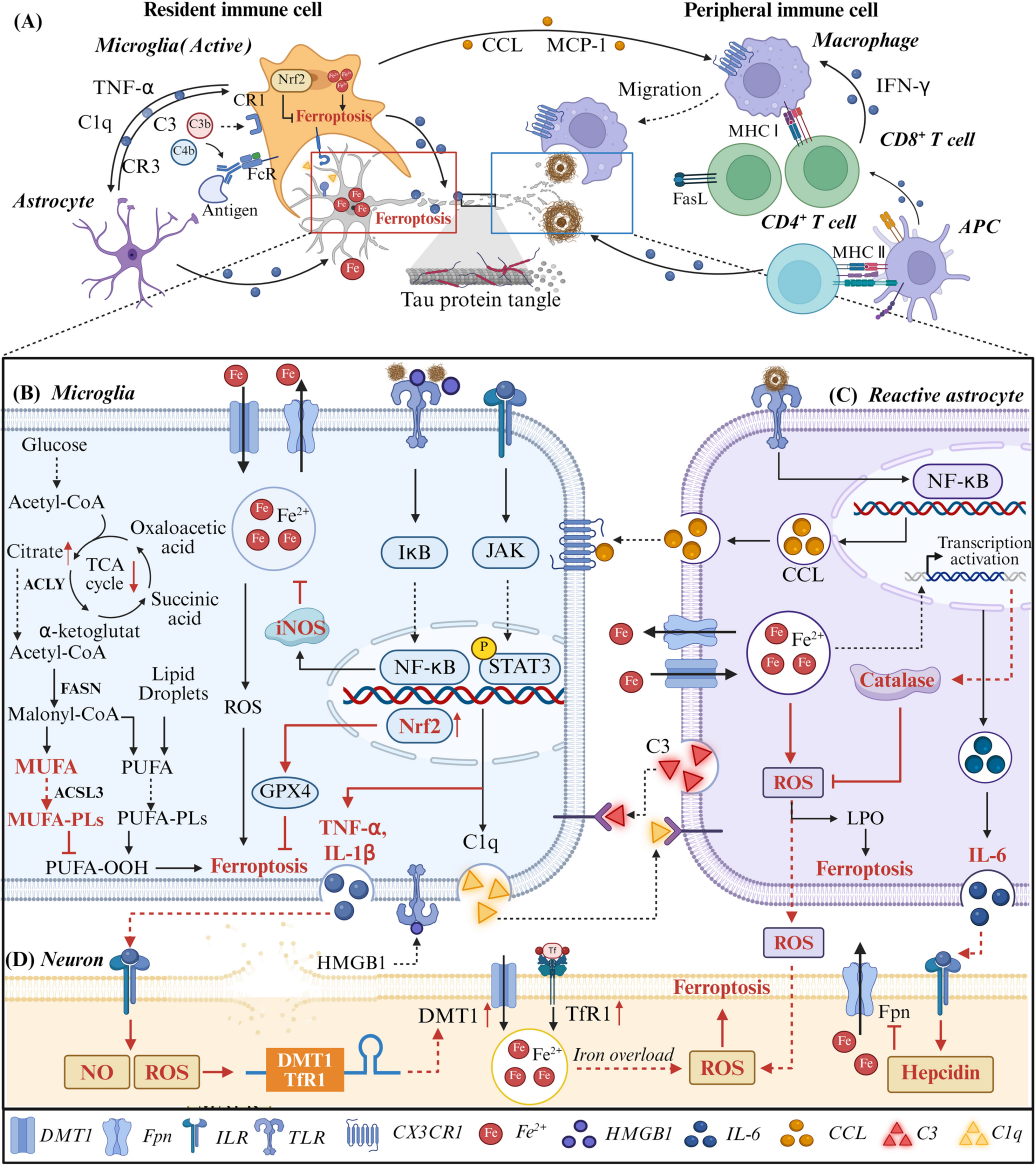
The crosstalk between ferroptosis and neuroinflammation in the pathogenesis of AD. **(A)** Above the figure is an overview diagram showing the interactions between central immune cells and peripheral immune cells in AD. Below, the focus is on the interaction relationships among neurons, astrocytes and microglia. **(B)** In microglial cells, Aβ and neuronal debris activate the NF-κB pathway, leading to the secretion of TNF-α, IL-1β and C1q. TNF-α and IL-1β binds to the surface receptors of neurons, promoting the synthesis of NO and ROS, increasing the synthesis of iron transport proteins DMT1 and TfR1, and causing iron overload and ferroptosis in neurons. Furthermore, in the context of AD, microglia cells exhibit iron accumulation, yet they possess strong resistance to ferroptosis. Firstly, NF-κB can upregulate the expression of iNOS and inhibit iron accumulation. Secondly, activated Nrf2 promotes the synthesis of GPX4 and inhibits lipid peroxidation. Thirdly, the alteration in the metabolic state of microglia may lead to an increase in MUFA synthesis, thereby reducing the production of PUFA-OOH. **(C)** Activated by microglia, reactive astrocytes express CCL and IL-6, thereby intensifying the inflammatory response. The IL-6 secreted by astrocytes acts on neurons, which can upregulate the expression of hepcidin and thereby inhibit the iron export protein Fpn. The ROS present within astrocytes can also directly enter neurons, exacerbating the oxidative stress state. **(D)** Therefore, under the influence of pro-inflammatory mediators secreted by glias, excessive iron accumulation occurs in neurons and the generation of ROS intensifies, eventually leading to ferroptosis. Conversely, the cellular debris and HMGB1 released during neuronal ferroptosis will further intensify the inflammatory response of immune cells such as microglia and astrocytes, forming a ferroptosis-immune interaction loop.

Within the AD brain, microglia adopt a predominantly pro-inflammatory phenotype, characterized by NF-κB-driven production of cytokines such as IL-1β, TNF-α, and IL-6, thereby amplifying neuroinflammation (65). Iron accumulation further reinforces this phenotype by promoting NADPH oxidase-dependent ROS generation and sustaining M1-like polarization (66, 67). Notably, despite their pro-oxidative milieu, microglia exhibit relative resistance to ferroptosis. This resistance is mediated by multiple mechanisms, including iNOS-dependent lipid radical scavenging, Nrf2-driven antioxidant responses (for example, GPX4 and glutathione synthesis), and metabolic reprogramming toward MUFA synthesis, which stabilizes membrane lipids. These layered defenses allow microglia to sustain inflammatory signaling while avoiding ferroptotic death (**Figure 3B**). In contrast, neurons are intrinsically more susceptible to ferroptosis due to their enrichment in polyunsaturated fatty acids and comparatively limited antioxidant capacity. Pro-inflammatory cytokines released by microglia, such as TNF-α and IL-1β, exacerbate this vulnerability by upregulating iron importers (TfR1 and DMT1) and increasing intracellular iron through ROS- and NO-dependent mechanisms (68, 69). This divergence in ferroptosis susceptibility between microglia and neurons represents a critical determinant of selective neuronal degeneration in AD (**Figure 3D**).

Astrocytes further modulate this ferroptosis–inflammation axis through bidirectional crosstalk with microglia and neurons. In response to Aβ, astrocytes activate NF-κB signaling and release complement component C3, which disrupts synaptic integrity via C3a receptor signaling (70). Concurrently, microglia-derived IL-1α, TNF-α, and C1q drive astrocytes toward a neurotoxic phenotype (71). These reactive astrocytes promote neuronal ferroptosis through multiple mechanisms, including IL-6-mediated activation of the JAK/STAT3 pathway, which induces hepcidin expression and suppresses ferroportin, thereby enhancing neuronal iron retention (72), as well as direct ROS release that accelerates lipid peroxidation (73). Intriguingly, astrocytes themselves exhibit adaptive resistance to oxidative stress by upregulating glutathione synthesis and antioxidant enzymes such as catalase (74), further underscoring the cell type-specific nature of ferroptosis regulation in the AD microenvironment (**Figure 3C, D**).

Beyond resident glial cells, peripheral immune components also contribute to the ferroptosis-inflammation interplay. BBB disruption, potentially driven by endothelial ferroptosis in response to Aβ, facilitates the infiltration of peripheral immune cells, including T lymphocytes (75, 76). Infiltrating CD4^+^ T cells can exacerbate pathology by secreting pro-inflammatory cytokines (for example, IFN-γ and TNF-α), which further activate microglia, impair BBB integrity, and promote neuronal ferroptosis through amplification of oxidative stress and iron dysregulation (**Figure 3A**) (77, 78). However, the role of T cells in AD remains controversial. While some studies report a correlation between T cell infiltration and disease severity, others observe minimal involvement or even context-dependent protective effects (79). These discrepancies likely reflect differences in disease stage, microenvironmental cues, and T cell subset composition.

Collectively, these findings highlight that ferroptosis in AD is highly cell type-specific and context-dependent, rather than a uniform pathological process. A major limitation of current studies is the reliance on bulk tissue analyses or simplified *in vitro* systems, which fail to capture the heterogeneity of cellular responses and intercellular interactions. Future investigations integrating single-cell and spatially resolved approaches will be essential to delineate how ferroptosis dynamically interacts with immune networks across disease progression, and to identify context-specific therapeutic windows.

### 4.2 PD

PD is a progressive neurodegenerative disorder affecting over 6 million individuals globally, clinically characterized by resting tremor, muscle rigidity, bradykinesia, and postural instability. Pathologically, PD is hallmarked by the selective loss of dopaminergic (DA) neurons in the substantia nigra pars compacta and the accumulation of Lewy bodies (80, 81). The underlying pathophysiology is multifactorial, involving mitochondrial dysfunction, lipid peroxidation, neuroinflammation, immune dysregulation, and dysregulated iron metabolism (82). Recent work has shown that microglia can promote neuronal ferroptosis via iron deposition, and that α-synuclein aggregation directly triggers ferroptosis (83, 84). These observations suggest an interaction between neuronal ferroptosis and immune cells in PD, although the precise mechanisms remain unclear.

**Figure 4:**
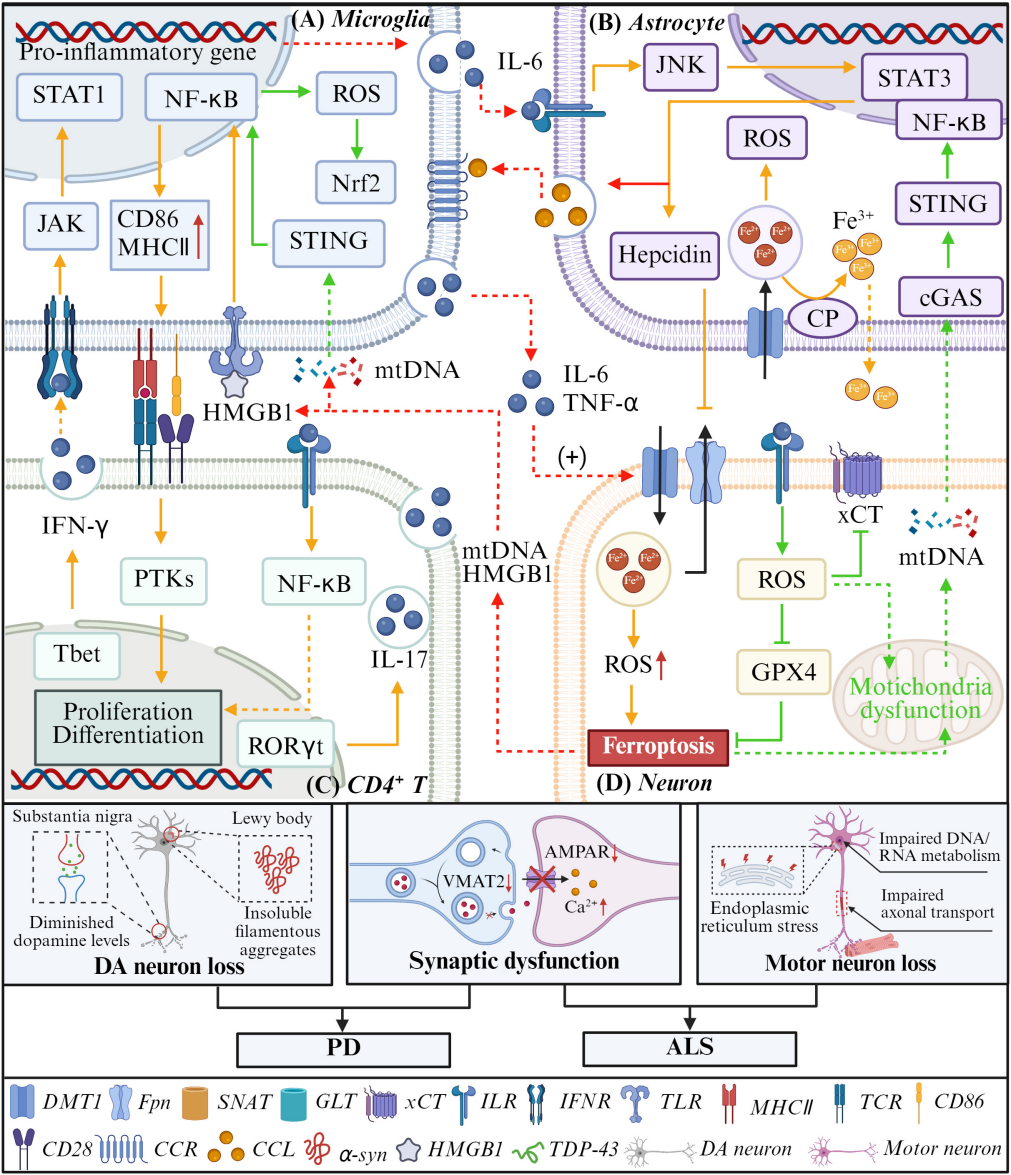
The mechanism of ferroptosis and immune cells interactions in PD and ALS. In PD (yellow and red arrows), due to iron deposition in dopamine neurons, ferroptosis occurs. **(A)** The ferroptotic neurons release HMGB1, which binds to the TLR4 receptor of microglia, activating NF-κB and inducing the release of pro-inflammatory factors. **(B)** IL-6 can activate the JAK/STAT3 pathway in astrocytes and promote the production of chemokines and hepcidin. **(D)** In addition, IL-6 and TNF-α could upregulate the expression of DMT1 in neurons, further promoting iron deposition. **(C)** Microglia can also act as antigen-presenting cells to activate T cells and promote their differentiation into Th1 and Th17 phenotypes, thereby amplifying the inflammatory response. In ALS (green and red arrows), because of the downregulation of neuronal system xc- and GPX4, intracellular ROS accumulation occurs, which subsequently triggers ferroptosis and mitochondrial damage. **(A)** The mtDNA released by mitochondrial damage can act on microglia and astrocytes, and through the cGAS/STING pathway, it upregulates NF-κB and promote neuroinflammation.

Mechanistically, exposure of microglia to pro-inflammatory stimuli (such as HMGB1, 4-HNE, and misfolded α-syn) induces microglial polarization from a surveillant M0 state to a deleterious M1 phenotype via activation of NF-κB and MAPK signaling pathways (10). This pro-inflammatory polarization not only augments the secretion of cytokines but also upregulates iron importers such as DMT1 and downregulates the iron exporter FPN1 in both microglia and neurons, thereby exacerbating iron accumulation and lipid peroxidation (**Figure 4A, D**) (69). Specifically, IL-6 activates the JAK/STAT3 pathway in astrocytes, inducing hepcidin production which binds and degrades FPN1, impeding iron efflux from neurons and sustaining intracellular iron overload (72). Moreover, α-syn aggregates and iron overload synergistically stimulate NOX complexes in microglia, elevating ROS generation and further compromising DA neuron viability (85). Interestingly, Kapralov AA et al. discovered through *in vitro* experiments that microglia deploy intrinsic ferroptosis resistance mechanisms by upregulating iNOS and downregulating 15-LOX (39). These adaptations help microglia survive oxidative environments while perpetuating neuroinflammation.

Astrocytes, key regulators of neuroinflammation and brain iron homeostasis, also contribute to PD pathogenesis. Activated by microglial cytokines and complement protein C1q, reactive astrocytes disrupt iron balance and induce neuronal ferroptosis (86). A central player in astrocytic iron regulation is ceruloplasmin (CP), a glycosylphosphatidylinositol-anchored ferroxidase on astrocyte surfaces that oxidizes Fe²^+^ to Fe³^+^, facilitating iron export and inhibiting Fe²^+^-induced lipid peroxidation (**Figure 4B**) (87, 88). Notably, PD patient substantia nigra samples reveal approximately 80% reduction in CP activity, correlating with aggravated iron deposition and DA neuron death. Conversely, experimental overexpression of CP in astrocytes restores iron homeostasis and suppresses ferroptosis. However, interestingly, an earlier study found that under oxidative stress conditions, astrocytes overexpress Nrf2 and enhance the survival ability of neurons by providing GSH to them (89). This suggests that the regulation of astrocytes on neuronal ferroptosis may be dependent on both the environment and time. In early PD, Nrf2-overexpressing astrocytes could enhance neuronal viability probably by an increased supply of GSH precursors. But chronic neuroinflammation drives reactive astrocytes to produce hepcidin, which impairs iron export and promotes ferroptosis.

Disruption of the neurovascular unit and BBB integrity during PD progression triggers recruitment of peripheral adaptive immune cells (90, 91). The infiltration of CD4^+^ and CD8^+^ T cells into the substantia nigra has been documented in both human patients and PD animal models (92, 93). Microglia present α-synuclein-derived peptides via MHC class II molecules, facilitating CD4^+^ T cell activation and differentiation into effector subsets (**Figure 4C**) (94, 95). However, the exact mechanisms by which infiltrating T cells modulate neuronal ferroptosis remain to be fully characterized. Emerging data suggest that CD4^+^ T cells may directly interact with astrocytes via immunological synapses, promoting astrocyte inflammatory phenotypes and potentially exacerbating neuronal injury, though further studies are needed to delineate these pathways (96).

While AD and PD share common features in microglial activation, iron accumulation, and T cell infiltration, key differences exist. In PD, astrocytic dysfunction plays a more prominent role, particularly the reduction in ceruloplasmin activity that exacerbates iron deposition. Additionally, α-syn aggregates directly promote ferroptosis by disrupting mitochondrial function and enhancing lipid peroxidation, whereas in AD, Aβ primarily acts through microglial activation. These differences suggest that ferroptosis inhibitors may need to be combined with disease-specific modulators (e.g., α-synuclein aggregation inhibitors in PD, Aβ clearance strategies in AD) for optimal efficacy.

### 4.3 ALS

ALS is a relentlessly progressive neurodegenerative disorder hallmarked by selective loss of upper and lower motor neurons, culminating in muscle atrophy and paralysis (97). Clinically, ALS patients exhibit elevated serum iron levels alongside heightened oxidative and inflammatory markers, correlating with profound motor deficits. In ALS murine models, intrinsic activation of microglia and astrocytes is accompanied by infiltration of peripheral lymphocytes and macrophages, as well as complement system upregulation. Notably, dysregulated iron homeostasis and neuronal ferroptosis induced by GPX4 inhibition have been documented (34, 98, 99). Interventions targeting ferroptosis attenuate neuroinflammation via reduced immune cell infiltration, while suppression of inflammation reciprocally diminishes neuronal ferroptosis. This implicates a tightly interconnected triad of ferroptosis, neuroinflammation, and immune cell dynamics in ALS pathogenesis. Nonetheless, the precise molecular regulatory circuits underpinning these interactions remain to be fully elucidated.

Neuroinflammation constitutes a defining hallmark of ALS progression, typified by a dysregulated balance between pro-inflammatory and anti-inflammatory mediators within microglia and astrocytes. In cerebrospinal fluid and peripheral blood from ALS patients, the level of pro-inflammatory cytokines (e.g., IL-6, IL-1β, and TNF-α) was elevated, and the concentrations of anti-inflammatory cytokines (e.g., IL-2 and IL-5) was decreased (100). Consequently, as the disease progresses, microglia shift to a predominantly pro-inflammatory phenotype. These activated microglia secrete cytokines and chemokines, which recruit additional resident and peripheral immune cells. At the same time, they exacerbate oxidative stress by driving ROS production in neurons and glial cells (101). ROS accumulation triggers the dissociation of Nrf2 from its cytoplasmic inhibitor Keap1, facilitating Nrf2 nuclear translocation. This event paradoxically leads to downregulation of the cystine/glutamate antiporter subunit SLC7A11 and GPX4 in motor neurons, thereby precipitating ferroptotic cell death (**Figure 4D**). Yang B et al. demonstrated that Nrf2 inactivation exacerbates ferroptosis in both human SOD1G93A ALS cell models and mouse models. Pharmacological activation of Nrf2 by RTA-408 restores the expression of SLC7A11 and GPX4 and elevates GSH levels. This intervention mitigates ferroptosis and thereby confers neuroprotection both *in vitro* and *in vivo* (98).

At the cellular level, ferroptotic motor neurons exhibit disrupted iron metabolism and excessive ROS, potentiating oxidative stress. In SOD1-mutant ALS mice, iron accumulation in microglia is associated with their polarization toward a pro-inflammatory M1 phenotype. This iron overload also promotes metabolic reprogramming toward glycolysis. Together, these changes amplify neuroinflammatory cascades and exacerbate motor neuron injury (32, 102). Furthermore, lipid peroxidation products released from ferroptotic neurons can inflict mitochondrial damage and provoke the release of mitochondrial DNA, which activates the cGAS/STING pathway in adjacent microglia and astrocytes, thereby upregulating transcription of pro-inflammatory cytokines (**Figures 4A, D**). Liddell et al. further reported that ferroptosis-induced stress in microglia precipitates an inflammatory cascade predominantly mediated by reactive astrocytes, culminating in non-cell-autonomous neuronal death (103).

In summary, ferroptosis and neuroinflammation constitute interdependent drivers of motor neuron degeneration in ALS, interconnected through a ROS-mediated feedback loop. Inflammatory cell-derived ROS potentiate neuronal ferroptosis by inhibiting Nrf2 activation and downregulating GPX4, while ferroptotic cells exacerbate neuroinflammation by fostering iron and ROS accumulation that promotes pro-inflammatory microglial polarization and cGAS/STING pathway activation.

Notably, immune cells promote neuronal ferroptosis via distinct core mechanisms across neurodegenerative diseases. In AD and PD, the focus lies on disrupting neuronal ferritin function and iron storage homeostasis, whereas in ALS, immune cells primarily target the neuronal antioxidant system (xCT and GPX4) to induce ferroptosis. This disease-specific divergence underscores the need for tailored therapeutic strategies. Despite these advances, detailed mechanistic insights into the molecular crosstalk between ferroptosis, neuroinflammation, and immune cell dynamics in ALS remain to be fully delineated.

### 4.4 MS

MS is a chronic immune-mediated disorder of the CNS, characterized by demyelination and progressive axonal degeneration. As an autoimmune disease, both innate and adaptive immune compartments orchestrate disease progression, with autoreactive T lymphocytes playing a pivotal role in mediating CNS inflammation and neurodegeneration (104). Emerging evidence implicates ferroptosis as a critical mechanism in MS pathogenesis. Siotto et al. reported altered expression of genes and proteins linked to neuronal iron metabolism and oxidative balance in MS patients, underscoring the disruption of iron homeostasis as a key pathological feature (105). These observations suggest that ferroptosis and immune dysregulation jointly contribute to MS pathology, although the mechanistic interplay between these processes remains incompletely understood.

**Figure 5:**
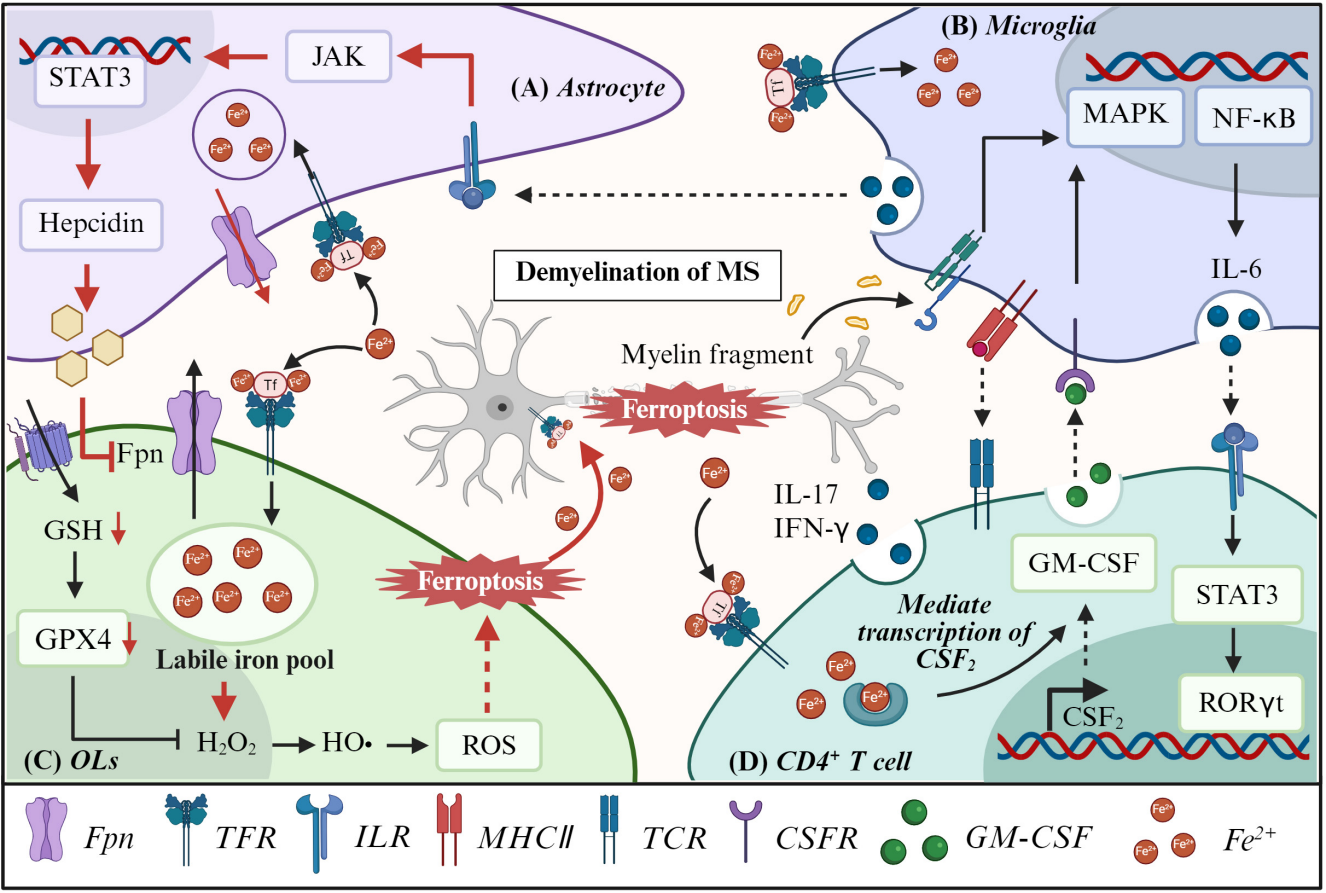
The interaction between ferroptosis and immune cells in MS. **(C)** As demyelination occurs, iron released from myelin fragment is imported into oligodendrocytes (OLs), which contributes to iron overload and ferroptosis of OLs. As a consequence, ferroptosis OLs releases intracellular iron into the extracellular space, where it can be taken up by neuron. This further exacerbated the process of neuronal ferroptosis and demyelination. **(B)** The fragments of demyelinated neurons bind to the surface receptors of microglia, activating the MAPK and NF-κB signaling pathways, and promoting the secretion of inflammatory factors such as IL-6. **(A)** On the one hand, IL-6 can activate the JAK/STAT3 pathway in astrocytes, promoting the synthesis and secretion of hepcidin. Then, hepcidin binds to Fpn in neurons and OLs, exacerbating their iron accumulation and ferroptosis. **(D)** On the other hand, IL-6 acts on CD4+ T cells, upregulates the expression of RORγT, and promotes their secretion of IL-17 and IFN-γ. Additionally, iron can be imported into T cells. In the cytoplasm, iron interacts with proteins and this complex then translocates to the nucleus to modulate the expression of csf2, which encodes for the protein GM-CSF. The release of GM-CSF from T cells can activate microglia and cause inflammation.

In MS, ferroptosis modulates inflammatory responses of immune cells, particularly CD4^+^ T cells. Duarte-Silva and colleagues demonstrated that dysregulated iron metabolism facilitates the differentiation of pathogenic T lymphocytes (106). Mechanistically, intracellular iron binds to the RNA-binding protein poly(rC)-binding protein, enhancing its stabilization of CSF2 mRNA, thereby promoting GM-CSF secretion. This cytokine milieu drives inflammatory activation of brain-resident immune cells, culminating in demyelination and tissue damage (107). Therapeutically, blockade of CD71-mediated iron uptake using anti-CD71 monoclonal antibodies attenuated experimental autoimmune encephalomyelitis (EAE), the murine model of MS, by reducing iron overload in CD4^+^ T cells and dampening their pathogenic differentiation (49). Complementary work by Jinyuan et al. revealed that ferroptotic neurons release molecules that activate T cells via the TCR signaling pathway, promoting Th1 and Th17 polarization and secretion of pro-inflammatory cytokines IFN-γ and IL-17, thereby exacerbating disease progression (**Figure 5D**) (108). Of note, in mechanism research, the EAE mouse model is the most commonly used animal model for MS. However, it is necessary to clearly point out the limitations of the EAE model. EAE is typically induced by two main approaches: active immunization with myelin antigens or adoptive transfer of autoreactive T cells (109). In contrast, human MS arises from complex interactions between genetic and environmental factors. Notably, human MS also exhibits more heterogeneous clinical manifestations and involves a broader spectrum of immune cells. Therefore, the conclusions drawn from EAE research should be cautiously extrapolated to human MS.

Beyond T cell modulation, B cells play a critical role in human MS pathogenesis that is not fully recapitulated in EAE models. Autoreactive B cells contribute to disease by secreting pathogenic autoantibodies, presenting antigens to T cells, and producing pro-inflammatory cytokines (e.g., BAFF, IL-6) that amplify the autoimmune cascade. Jelcic I et al. found that depletion of B cells *in vitro* and therapeutically *in vivo* by anti-CD20 effectively reduces T cell autoproliferation, indicating that pathogenic B-T cell interactions is closely related to the pathogenesis of MS (110). This clinical relevance is highlighted by the therapeutic success of anti-CD20 monoclonal antibodies (111). Rituximab, a chimeric anti-CD20 antibody, was the first to show efficacy in reducing relapse rates and new inflammatory lesions in relapsing-remitting MS patients in a phase II trial (112). These clinical successes underscore the need to integrate B cell biology into the understanding of MS ferroptosis-immune crosstalk. However, the studies on B cells in MS are insufficient. Future studies investigating B cell-ferroptosis interactions may uncover novel therapeutic opportunities.

In addition, ferroptosis impacts CNS-resident cells integral to MS pathology. Oligodendrocytes (OLs), responsible for myelin synthesis, exhibit heightened vulnerability to ferroptosis owing to their substantial iron content (113). Li X et al. documented downregulation of system Xc^-^ and GPX4 in the spinal cords of EAE mice, accompanied by increased lipid peroxidation and ROS accumulation within OLs; ferrostatin-1 administration partially rescued these changes, affirming the involvement of ferroptosis in OL-mediated demyelination (114). Furthermore, astrocyte-oligodendrocyte crosstalk critically influences iron homeostasis: iron uptake by astrocytes induces hepcidin secretion, which downregulates Fpn on OLs, promoting iron retention and ferroptosis (**Figure 5A, C**) (106, 115). Iron released from ferroptotic OLs accumulates extracellularly, facilitating microglial and macrophage iron uptake, driving Fenton reactions, oxidative stress, and neuroinflammation that potentiate neurodegeneration (116, 117).

In summary, ferroptosis-immune crosstalk in MS involves T cell iron metabolism driving pathogenic differentiation, B cells amplifying autoimmunity, and oligodendrocyte ferroptosis directly causing demyelination. Unlike ALS, where immune responses exhibit stage-dependent duality, MS shows more consistent pathogenicity across stages, yet shifts from peripheral to CNS-compartmentalized inflammation. These complexities, coupled with model limitations, underscore the need for cell-type-selective and stage-adapted therapeutic strategies.

### 4.5 Stroke

Stroke ranks as the second leading cause of mortality worldwide and is associated with substantial disability and fatality rates (118, 119). Clinically, strokes are classified into ischemic stroke (IS) and hemorrhagic stroke, with IS accounting for approximately 87% of cases (119). The pathophysiology of IS involves complex and interrelated processes, including oxidative stress, regulated cell death, mitochondrial dysfunction, and neuroinflammation (120). Previous studies have underscored a tightly intertwined relationship between immune cells, neuroinflammation, and ferroptosis, whose crosstalk critically influences post-stroke outcomes (121, 122). However, the discrete and interactive roles of ferroptosis and inflammation in IS pathogenesis remain incompletely elucidated.

**Figure 6:**
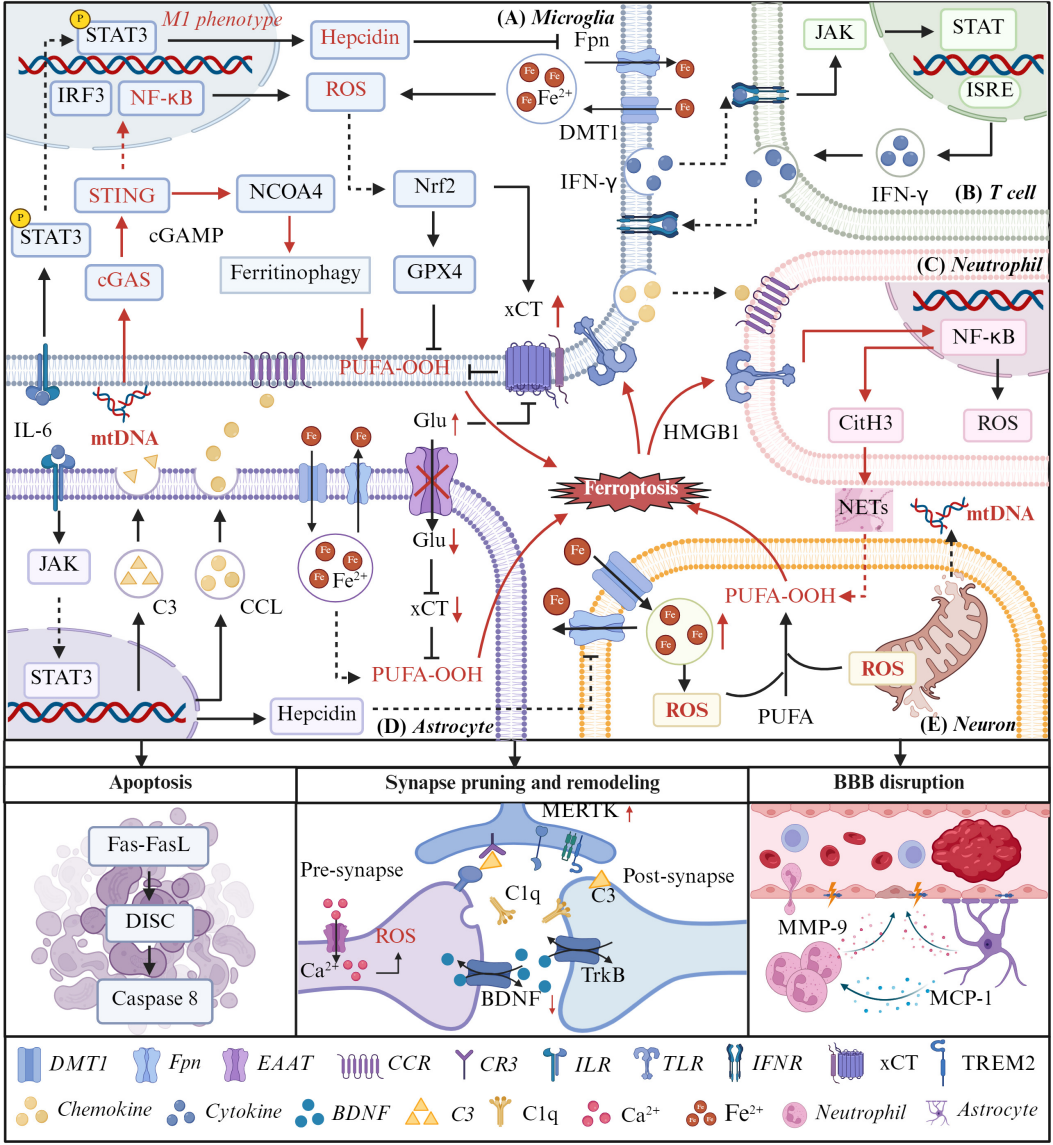
The interplay between ferroptosis and neuroinflammation in the pathogenesis of IS. **(E)** Ischemia injury causes the rupture of neuronal mitochondria, accumulation of iron within cells, and increased production of ROS. Eventually, ferroptosis occurs, releasing HMGB1 and mtDNA. **(A)** Various dsDNA molecules derived from deceased cells or cellular organelles can participate in inflammatory responses and ferroptosis by activating the cGAS/STING pathway and NF-κB in microglia. Subsequently, activated STING upregulates nuclear receptor coactivator 4(NCOA4) expression, leading to ferritinophagy and a surge in ROS production via the Fenton reaction. Furthermore, because of the activation of signals such as NF-κB and STAT3, microglia exhibit a M1 pro-inflammatory phenotype, increasing the synthesis of ROS. Due to the excessive accumulation of ROS, Nrf2 is activated in microglia and exerts a regulatory role in ferroptosis. **(B)** The IFN-γ secreted by microglia can activate the JAK/STAT pathway of T cells, thereby amplifying the inflammatory response. **(C)**Neutrophils produce ROS to increase neuronal oxidative stress, and on the other hand, activated by HMGB1 released from ferroptosis cells neutrophils release NETs, eventually leading to neuronal ferroptosis. **(D)** IL-6 activates the JAK/STAT3 pathway in astrocytes, thereby promoting the secretion of hepcidin by these cells and inhibiting the surface Fpn iron transporter on neurons. Meanwhile, under inflammatory conditions, the glutamate transporter EAAT on the surface of astrocytes becomes inactive, resulting in the accumulation of extracellular glutamate. High levels of glutamate will inhibit the surface system xc- of microglia, reduce their antioxidant capacity and promote ferroptosis.

Ischemia-reperfusion injury precipitates a cascade of detrimental events, including diminished cerebral perfusion, ATP depletion, glutamate excitotoxicity, and excessive intracellular Ca^2+^ accumulation, culminating in mitochondrial dysfunction. Damaged mitochondria release aberrant mtDNA, which can trigger innate immune pathways, while concomitant ROS overproduction promotes lipid peroxidation and ferroptotic death in neurons (**Figure 6E**) (123, 124). Subsequently, the signaling molecules released by neurons (e.g. HMGB1 and lipid peroxidation products) act on microglia, activating signaling pathways such as NF-κB and cGAS-STING (44). Among the signaling pathways linking ferroptosis to inflammation, the cGAS-STING pathway has emerged as a key mediator. Ma X et al. pointed that oxidative DNA damage post-IS activates cGAS-STING in microglia, promoting NCOA4-mediated ferritinophagy and thereby increasing intracellular labile iron and ROS accumulation (**Figure 6A**) (125, 126). Intriguingly, Tao et al. demonstrated that excessive ROS could inhibit the cGAS-STING axis and downregulate IFN-I expression, indicating that ROS may play a negative feedback regulatory role in this pathway (127). This also suggests that the cGAS-STING signaling pathway may have a time-dependent regulatory role in stroke, but the specific pattern of changes remains unclear. Therefore, future research should further delve into exploring the temporal variation patterns of this pathway.

In addition to activating microglia cells, neuronal ferroptosis orchestrates peripheral immune cell recruitment, aggravating neuroinflammation via HMGB1 and neutrophil NET formation. In permanent MCAO models, serum HMGB1 levels surge rapidly after ischemia. HMGB1 binds to CXCR4 and TLR4 receptors on neutrophils, triggering NET formation. Within hours after MCAO, these activated neutrophils infiltrate the brain parenchyma through leptomeningeal routes, amplifying neuroinflammation and exacerbating neuronal ferroptosis (41). This evidence delineates a deleterious feed-forward loop wherein neuronal ferroptosis and NETosis mutually exacerbate ischemic injury, with HMGB1 acting as a pivotal molecular nexus (**Figure 6C**).

Beyond the interaction between neuronal ferroptosis and immune cells, microglial polarization further modulates the ferroptosis-inflammation nexus. OGD/R models reveal an imbalance favoring pro-inflammatory M1 microglia, alongside activation of the IL-6/STAT3 and BMP6/SMADs signaling cascades. This promotes secretion of inflammatory cytokines and upregulation of hepcidin, initiating Fenton chemistry and neuronal ferroptosis (128). Furthermore, the pro-inflammatory factors secreted by M1 microglia can act on other immune cells (such as astrocytes and T cells), thereby amplifying the inflammatory response, intensifying oxidative stress and causing neuronal ferroptosis (**Figure 6B**). Among them, astrocytes activated by microglia cells exert dual pro-ferroptotic effects. On the one hand, they secrete hepcidin, which inhibits the function of Fpn in neurons, thereby promoting iron accumulation and ferroptosis. On the other hand, inactivation of the glutamate transporter EAAT in astrocytes impairs extracellular glutamate clearance, leading to glutamate accumulation in the extracellular space. Excess glutamate subsequently inhibits xCT activity on the surface of microglia and neurons. Reduced xCT activity weakens cellular antioxidant capacity and further increases vulnerability to ferroptosis (**Figure 6D**) (129).

In summary, stroke exemplifies the acute manifestation of ferroptosis-immune crosstalk, characterized by rapid neuronal ferroptosis, cGAS-STING-mediated microglial activation, and NETosis-driven neutrophil infiltration. While sharing mechanistic pathways with neurodegenerative diseases and glioma, stroke is distinguished by its acute onset, short therapeutic window, and the urgent need for interventions that can be administered within hours. Future therapeutic strategies should prioritize rapid BBB-penetrating ferroptosis inhibitors and stage-specific interventions that block acute damage without compromising long-term recovery.

### 4.6 Glioma

Glioma, the most common primary tumor of the CNS, encompasses a spectrum of malignancies, with glioblastoma multiforme representing the most aggressive and lethal subtype (130, 131). Despite multimodal therapies including temozolomide-based chemotherapy and emerging immunotherapies, prognosis remains dismal, largely attributed to the highly immunosuppressive tumor microenvironment (132, 133). Emerging evidence identifies ferroptosis as a key regulator of glioma progression and immune landscape remodeling (134, 135).

**Figure 7:**
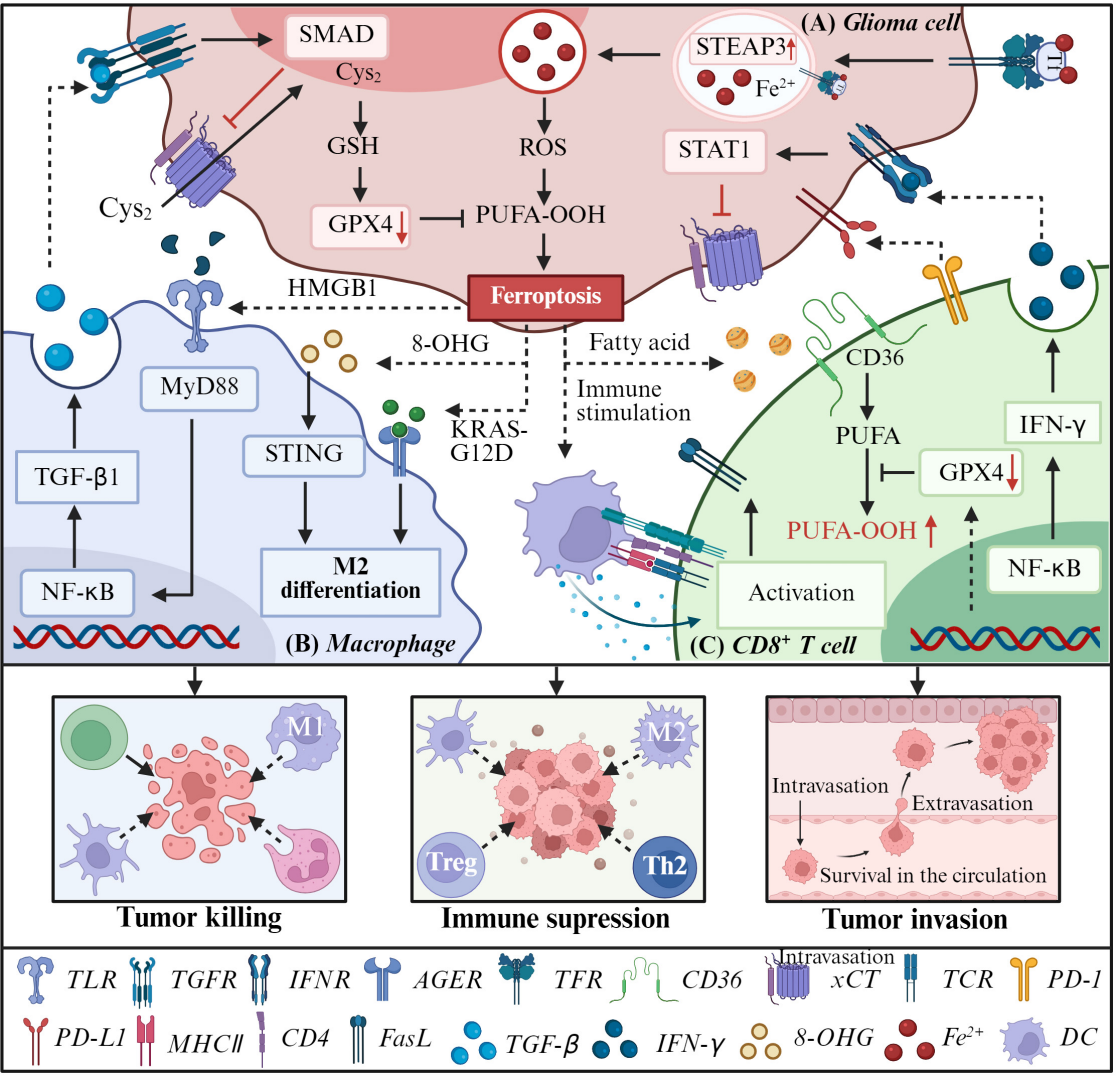
Role of ferroptosis in glioma immune microenvironment. **(A)** In glioma cells, the upregulation of TfR and STEAP3 protein increases iron intake and accumulation. Meanwhile, CD8+ T cells release IFN-γ to activate IFNR (which inhibits SLC7A11 transcription through STAT1) to promote glioma cell ferroptosis. TGF-β1 released by macrophages induces the downregulation of Xc- system mediated by SMAD proteins, thereby triggering lipid peroxidation and ferroptosis via the GSH-GPX4 axis. In turn, ferroptotic glioma cells release DAMPs (such as HMGB1 and AA) to promote the recruitment and activation of immune cells. However, several moleculars released by glioma cells affect the function of macrophage and T cells. **(B)** In macrophage, KRAS-G12D binds AGER on the cell surface of macrophages and 8-OHG activates STING proteins to trigger M2 macrophage polarization, which might limit its antitumor ability. **(C)** While in T cells, increased intake of fatty acids mediated by CD36 and decreased expression of GPX4 lead to excessive accumulation of lipid peroxides in cells, which promote ferroptosis of T cells and exert an immunosuppressive effect.

As shown in **Figure 7A**, ferroptotic glioma cells release DAMPs such as HMGB1, oncogenic KRAS-G12D, and 8-OHG, which function as chemotactic signals to recruit and activate immune cells for phagocytic clearance (136, 137). Notably, HMGB1 liberated from ferroptotic tumor cells activates dendritic cells, promoting their migration to cervical lymph nodes and priming systemic cytotoxic CD8^+^ T cell responses against glioma antigens (138). Subsequent IFN-γ secretion by activated CD8^+^ T cells engages IFN receptors on glioma cells, triggering downregulation of cystine/glutamate antiporter subunits SLC3A2 and SLC7A11 (**Figure 7C**). This impairs cystine uptake, exacerbates lipid peroxidation, and potentiates ferroptosis in tumor cells, establishing a positive feedback loop reinforcing antitumor immunity. Complementing this, ferroptosis induces translocation of calreticulin, an endoplasmic reticulum chaperone, to the tumor cell surface, serving as an “eat-me” signal that promotes phagocytosis and antigen presentation, thereby enhancing adaptive immune activation (139, 140). However, ferroptosis also paradoxically contributes to the immunosuppressive milieu. Liu et al. proposed that despite initial immune activation, persistent ferroptosis does not overcome tumor immune evasion. This may be explained by the meso-immune tidal model that is characterized by concurrent high expression of co-stimulatory and co-inhibitory immune checkpoints that sustain a suppressed immune phenotype in gliomas (141, 142).

Ferroptosis significantly modulates macrophage polarization within the glioma TME. Co-culture experiments demonstrate that ferroptosis inhibition via Fer-1 increases pro-inflammatory M1 macrophages while decreasing immunosuppressive M2 macrophages. Conversely, ferroptosis induction promotes macrophage chemotaxis and M2 polarization, which supports tumor progression through secretion of anti-inflammatory cytokines, extracellular matrix remodeling, and suppression of T cell function (143). Mechanistically, KRAS-G12D released from ferroptotic tumor cells activates RAGE on macrophages, driving M2 polarization and downstream immunosuppressive signaling cascades (**Figure 7B**) (144).

CD8^+^ T lymphocytes are pivotal for antitumor immunity but exhibit metabolic vulnerability to ferroptosis within the TME. GPX4-deficient CD8^+^ T cells show heightened ferroptosis sensitivity and impaired cytotoxicity (140). Tumor-infiltrating CD8^+^ T cells accumulate lipid peroxidation products absent in lymph node-resident T cells (145), and CD36-mediated uptake of oxidized lipids exacerbates ferroptosis, compromising antitumor function (146). In contrast, Tregs resist ferroptosis via upregulated GPX4 expression, perpetuating immune suppression (147). This differential susceptibility creates an immunosuppressive imbalance that favors tumor progression (**Figure 7C**). To overcome this hurdle, approaches that selectively induce ferroptosis in tumor cells while sparing T cells are needed. Promising strategies include nanoparticle-based delivery, CAR-T cells engineered with enhanced GPX4 expression, and CD36 blockade to prevent T cell lipid peroxidation. However, the efficacy and safety of these approaches require validation in rigorous preclinical and clinical studies.

Preclinical studies have suggested synergy between ferroptosis induction and immune checkpoint blockade. In glioma models, combining ferroptosis inducers (e.g., erastin) with anti-PD-L1 antibodies reprograms tumor-associated macrophages toward an M1-like phenotype, enhances T cell infiltration, and improves therapeutic efficacy (141, 148). The rationale is compelling. Ferroptosis-induced DAMPs prime antitumor T cell responses, while checkpoint inhibitors relieve T cell suppression (149). However, clinical translation will require careful optimization of timing and dosing to maximize synergy while minimizing toxicity.

This therapeutic exploitation of ferroptosis in glioma stands in stark contrast to neurodegenerative diseases discussed earlier in this review. In AD, PD, ALS, and MS, ferroptosis is uniformly detrimental, driving neuronal and oligodendrocyte loss while amplifying neuroinflammation. Thus, therapeutic strategies should aim to inhibit ferroptosis. In glioma, however, ferroptosis represents a therapeutic vulnerability. In glioma, however, ferroptosis represents a therapeutic vulnerability. Inducing ferroptosis in tumor cells enhances immunogenic cell death and primes antitumor immunity. This fundamental duality underscores the context-dependent nature of ferroptosis-immune crosstalk and the need for disease-specific therapeutic strategies.

## 5 Clinical therapeutic strategies for ferroptosis and immune cell interactions

The interplay between ferroptosis and immune cells constitutes a critical regulatory axis in neuropathological disorders, integrating immune cell polarization (e.g., microglia, dendritic cells, T cells), the modulation of immune responses, and the shaping of the local immune microenvironment. Therapeutically, manipulating ferroptosis can recalibrate immune cell function, enhance protective immune responses, and ameliorate neurodegeneration, neuroinflammation, and glioma progression. Conversely, targeting immune cells can attenuate neuroinflammatory cascades, limit lipid peroxidation and ferroptotic neuronal death, and thereby promote an immune-permissive microenvironment that favors neuronal survival and functional recovery. Collectively, interventions directed at ferroptosis, immune cells, or their reciprocal interactions offer a mechanistic framework for the prevention, diagnosis, and treatment of neuropathological injuries and CNS diseases (**Table 2**).

**Table 2:** Clinical therapeutic strategies for ferroptosis and immune cells interactions.

Therapeutic strategies	Compounds	Chemical structure	Target	Effects	Clinical approval status	Underlying mechanisms	Diseases	References
Ferroptosis inhibitor	DFO	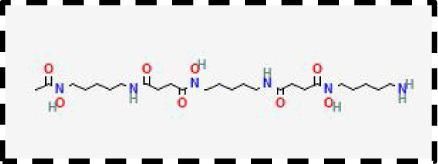	Iron metabolism	Suppress neuroinflammation; Improve cerebrovascular function	Phase II completed (ICH, TBI)	Iron chelator	Ischemic stroke, Aneurysmal subarachnoid hemorrhage; Alzheimer’s disease	(193–195)
	DFP	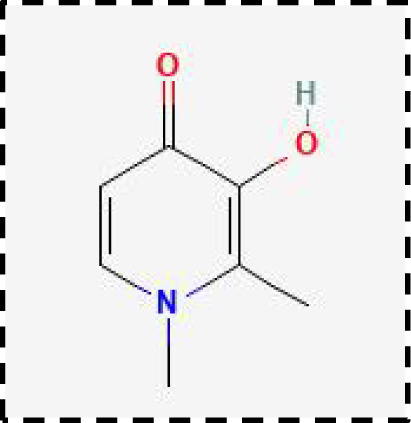	Iron metabolism	Reduce neuronal death	Phase II completed (PD, MS)	Iron chelator	Friedreich’s ataxia; Neurodegeneration with brain iron accumulation; Parkinson’s disease; Multiple sclerosis; Motor neuron disease	(196, 197)
	DFX	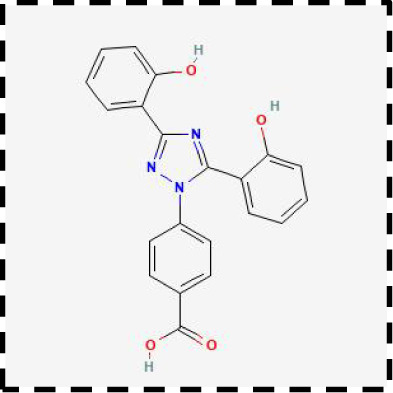	Iron metabolism	Reduch neuronal death; Prevent perihematomaledema	Preclinical	Iron chelator	Spinal cord injury; Hemorrhagic stroke; Traumatic brain injury	(198–200)
	Ferristatin II	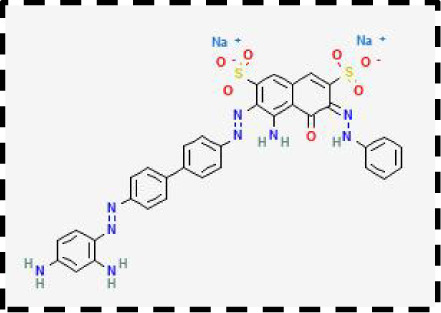	Iron metabolism	Attenuate neuronal injury and neurodegeneration	Preclinical	TfR1 inhibitor	Traumatic brain injury	(201)
	Edaravone	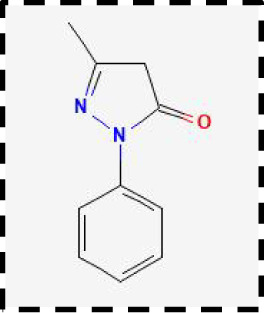	Antioxidant system	Reduce neuronal death	Approved (ALS, stroke)	Radical-trapping antioxidants	Acute ischemic stroke; Amyotrophic lateral sclerosis; Intracerebral haemorrhage	(202–204)
	Ferrostatin-1	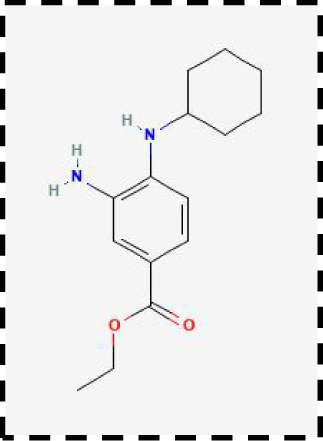	Antioxidant system	Improve neuron survival and scar formation	Preclinical	Radical-trapping antioxidants	Spinal cord injury; Intracerebral hemorrhage; Optic neuropathies; Parkinson’s disease; Huntington’s disease	(205–208)
	Liproxsatain-1	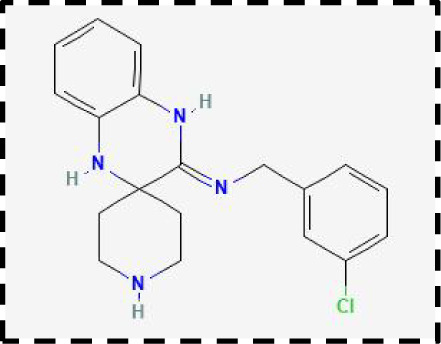	Antioxidant system	Reduce neuronal death and neuroinflammation	Preclinical	Radical-trapping antioxidants, inhibit the activation of microglia and serection of pro- inflammatory factor	Subarachnoid Hemorrhage; Ischemic stroke; Parkinson’s disease	(72, 160, 209)
Ferroptosis inducer	Sulfasalazine	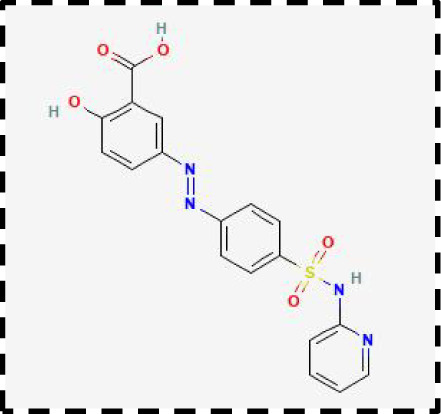	Antioxidant system	Inhibit tumor proliferation; Prevent seizures	Phase I/II terminated (GBM)	SLC7A11 inhibitor	Glioma; Glioblastoma; Recurrent glioblastoma	(172, 210)
	Erastin	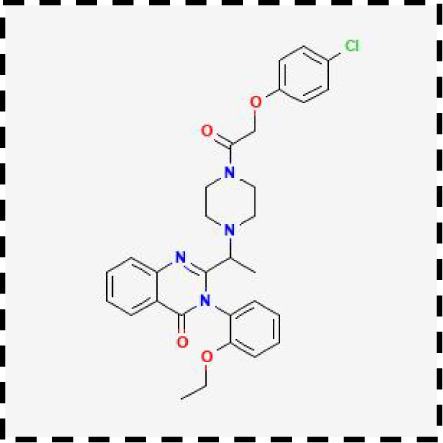	Antioxidant system	Inhibit tumor proliferation	Preclinical	SLC7A11 inhibitor	Meningioma, glioma	(164)
	Sorafenib	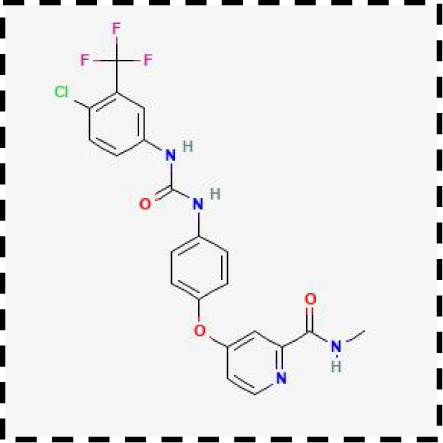	Antioxidant system	Inhibit tumor proliferation	Approved (HCC, RCC); Phase I (brain mets)	SLC7A11 inhibitor	Brain metastases	(171)
	RSL3	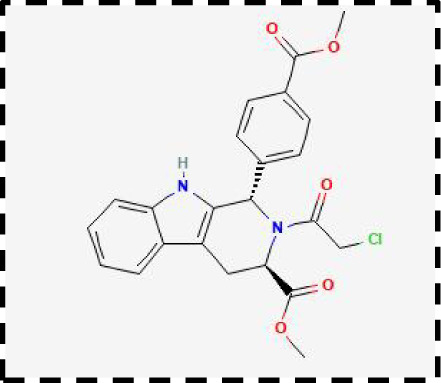	Antioxidant system	Inhibit tumor proliferation	Preclinical	GPX4 inhibitor	Glioma; Fibrosarcoma	(165, 211)
	FIN56	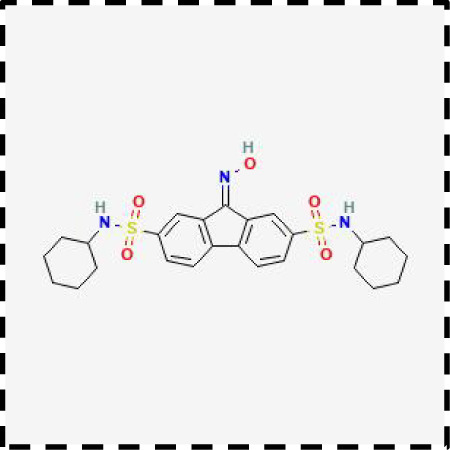	Antioxidant system	Inhibit tumor proliferation	Preclinical	GPX4 inhibitor	Glioblastoma	(167)
Immune microenvironment modulators	Rehmannioside A	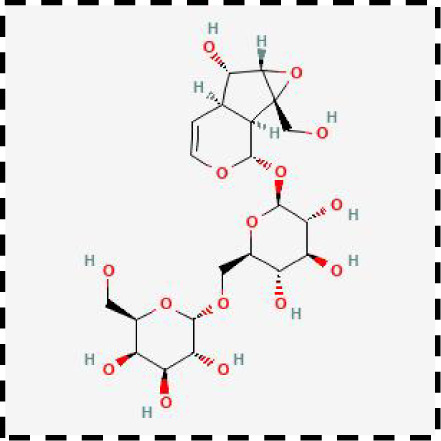	Antioxidant system; Neuroinflammation	Recude neuronal death and neuroinflammation	Preclinical	Activate PI3K/AKT/ Nrf2 and SLC7A11/GPX4 signaling pathway; Inhibit NF-κB and MAPK pathways	Cerebral ischemia; Depression; Spinal cord injury	(212, 213)
	Icariside II	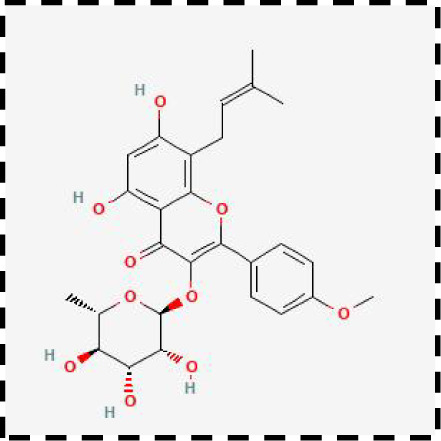	Antioxidant system; Neuroinflammation	Reduce neuronal death and neuroinflammation	Preclinical	Activate Nrf2- mediated OXPHOS/NF-κB/ ferroptosis axis and Nrf2/HO-1 pathway; Inhibit TLR4/MyD88/NF-κB pathway	Ischaemic stroke; Parkinson’s disease; Cerebral ischemia- reperfusion injury	(214–216)
	Forsythoside A	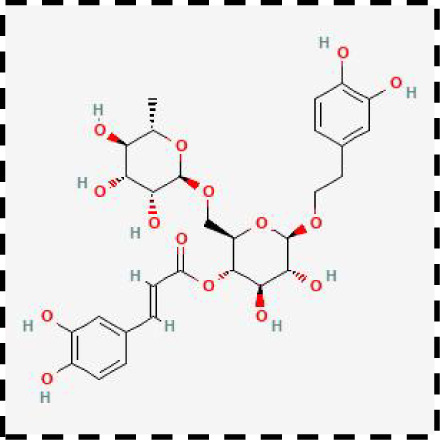	Antioxidant system; Neuroinflammation	Reduce neuronal death and neuroinflammation; Improve cognitive impairment	Preclinical	Activate Nrf2/GPX4 axis; Inhibit NF-κB pathway	Alzheimer’s disease; Parkinson’s disease; Ischemic stroke; Traumatic brain injury	(175, 217)
	Quercetin	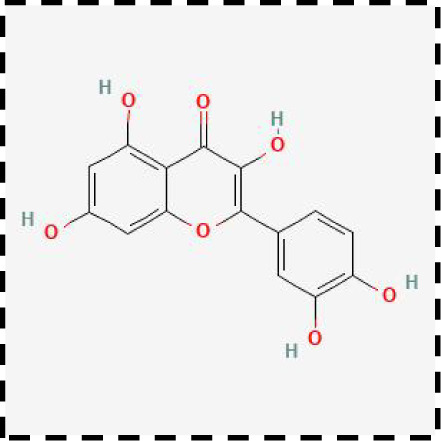	Antioxidant system; Neuroinflammation	Reduce neuronal death and neuroinflammation	Phase I/II	Activate SIRT1/Nrf2/SLC7A11/GPX4 pathway and AMPK expression; Inhibit JNK and PI3K/AKT/NF-κB pathway	Epilepsy; Stroke; Alzheimer’s disease; Parkinson’s disease; Spinal cord injury	(218–220)
	Astragaloside IV	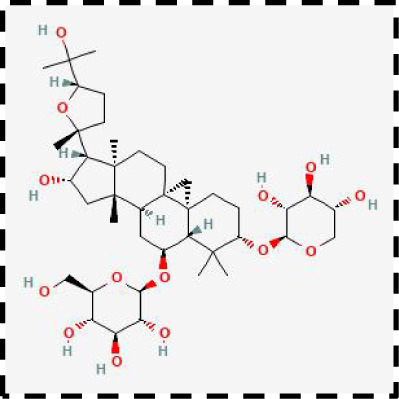	Antioxidant system; Neuroinflammation	Reduce neuronal death and neuroinflammation	Preclinical	Activate P62/Keap1/Nrf2 pathway	Ischaemic stroke; Alzheimer’s disease	(221, 222)
Combination therapeutic strategy	Ferrostatin-1+Anti-PDL1 antibodies	/	Immunosuppressive checkpoint; Ferroptosis	Enhance the sensitivity of glioma cells to immune checkpoint therapy; Increase the infiltration of CD3^+^ T cell	Preclinical	Radical-trapping antioxidants; Activate cellular immunity	Glioma	(141)
	Temozolomide+Haloperidol	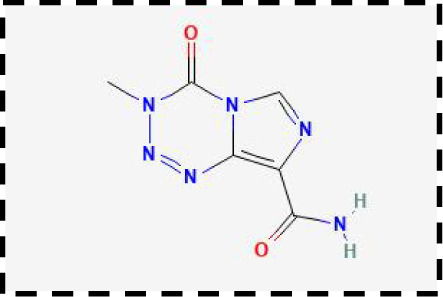 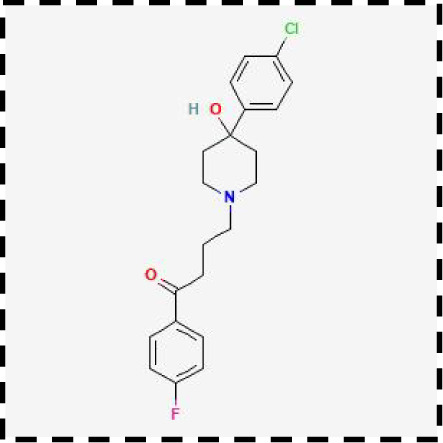	Glioma cells; Ferroptosis	Overcome chemoresistance of glioma	Preclinical	Activate cAMP/PKA pathway; Trigger endoplasmic reticulum stress and induce ferroptosis of glioma cells	Glioma	(223)
	Minocycline+NAC	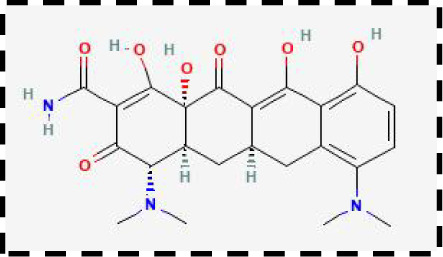 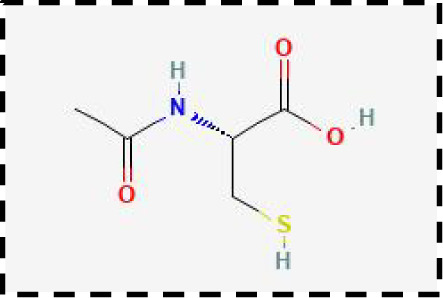	Ferroptosis; Neuroinflammation	Reduce neuronal death and neuroinflammation	Preclinical	Suppress the activation of microglia; Inhibit mitochondrial cytochrome C release; Regulate GSH synthesis	Traumatic brain injury	(184)
	SRF@FeIIITA	/	Glioma cells; Ferroptosis	Inhibit tumor proliferation	Preclinical	Increase the level of free iron in glioma cells; Inhibit the activity of SLC7A11	Glioma	(185)
	T-LNPs-Fer1	/	Ferroptosis	Penetrate the BBB; Enhance the neuroprotective effect of Fer-1 on damaged neurons	Preclinical	Radical-trapping antioxidants	Cerebral ischemic stroke	(189)

### 5.1 Ferroptosis inhibitors

Ferroptosis inhibitors comprise iron chelators, pathway-targeted modulators, and antioxidants, which collectively suppress iron-dependent lipid peroxidation and neuronal cell death. Among these, iron chelation represents the most clinically advanced strategy. Agents such as deferoxamine (DFO), deferiprone (DFP), and deferasirox sequester labile iron, thereby limiting Fenton chemistry and downstream lipid peroxidation.

DFO, widely used for systemic iron overload, has demonstrated robust neuroprotective effects in preclinical models of cerebral ischemia and intracerebral hemorrhage, in part through activation of the Nrf2-TXNRD1 axis and attenuation of ferroptotic injury (150, 151). Importantly, DFO has progressed to phase II clinical trials in intracerebral hemorrhage and traumatic brain injury, where it exhibited an acceptable safety profile, although its clinical efficacy remains modest and optimal dosing regimens are yet to be defined (152). Similarly, DFP has shown efficacy in reducing brain iron deposition and myelin damage in patients with multiple sclerosis (153). However, results from the FAIRPARK-II phase II trial in Parkinson’s disease revealed that, despite measurable reductions in iron load, clinical benefits were limited, suggesting that iron chelation alone may be insufficient to halt disease progression (154). Moreover, systemic iron depletion raises safety concerns, including anemia and neuromuscular side effects, underscoring the need for more targeted approaches (155, 156).

Antioxidant-based strategies constitute another major class of ferroptosis inhibitors. As a central regulator of ferroptosis, GPX4 detoxifies lipid hydroperoxides, and its activation is critical for maintaining redox homeostasis. Selenium supplementation, including sodium selenite and selenocompounds such as SeMet, supports GPX4 biosynthesis and has shown protective effects in models of ischemia-reperfusion injury by attenuating oxidative stress and mitochondrial dysfunction (157). In parallel, radical-trapping antioxidants (RTAs), including ferrostatin-1 (Fer-1) and liproxstatin-1 (Lip-1), inhibit lipid peroxidation independently of GPX4 (158). While these agents demonstrate potent neuroprotection in preclinical models, their clinical translation is limited by poor pharmacokinetic properties and insufficient BBB penetration (159, 160).

Notably, edaravone represents a successful example of translational advancement. By scavenging free radicals and modulating the Nrf2/HO-1/GPX4 axis, edaravone has been approved for ALS in the United States and Japan and is widely used for acute ischemic stroke, providing clinical proof-of-concept that targeting oxidative stress and ferroptosis is therapeutically viable (161, 162).

### 5.2 Ferroptosis inducers

In contrast to neuroprotection, ferroptosis induction has emerged as a promising strategy for targeting therapy-resistant tumors, particularly gliomas. Ferroptosis inducers act by promoting intracellular iron accumulation, enhancing lipid peroxidation, or disabling antioxidant defenses. Given that tumor cells often exhibit iron dependency and elevated redox buffering capacity, they are particularly susceptible to ferroptotic stress.

Classical ferroptosis inducers, including erastin, RSL3, and FIN56, have demonstrated robust anti-tumor efficacy in preclinical studies. Erastin inhibits the system Xc^-^ cystine/glutamate antiporter, thereby depleting intracellular glutathione and triggering ferroptosis. However, its poor solubility and unfavorable pharmacokinetics have hindered clinical translation (163, 164). RSL3 directly inhibits GPX4, inducing ferroptosis in glioma and fibrosarcoma models, but its lack of selectivity raises concerns regarding off-target toxicity in normal tissues (140, 165, 166). Similarly, FIN56 promotes GPX4 degradation and activates squalene synthase to drive lipid peroxidation, yet remains confined to preclinical investigation (167, 168).

Among clinically relevant agents, sorafenib, a multi-kinase inhibitor approved for hepatocellular carcinoma and renal cell carcinoma, has been shown to induce ferroptosis by inhibiting system Xc^-^ and depleting glutathione (169, 170). In early-phase clinical studies for brain tumors, including trials combining sorafenib with radiotherapy, modest therapeutic effects have been observed, suggesting limited efficacy as a monotherapy (171). Likewise, sulfasalazine, another system Xc^-^ inhibitor, entered clinical trials for recurrent glioblastoma but was terminated prematurely due to insufficient efficacy and dose-limiting toxicity (172). These findings highlight a key challenge in ferroptosis induction, namely that achieving tumor-selective cytotoxicity without compromising normal cellular antioxidant defenses is difficult.

Collectively, current clinical and preclinical evidence underscores both the promise and limitations of ferroptosis-targeted therapies. While iron chelators and antioxidants such as DFO, DFP, and edaravone have demonstrated translational potential, their therapeutic efficacy is often partial, and long-term safety profiles remain to be fully established. Conversely, most ferroptosis inducers are still confined to preclinical stages, with only a limited number progressing to early-phase clinical evaluation. Several key barriers hinder clinical translation. First, inadequate BBB penetration restricts the efficacy of many ferroptosis modulators in CNS diseases. Second, the lack of cell-type specificity raises concerns regarding off-target toxicity, particularly for GPX4 inhibitors and systemic iron chelators. Third, insufficient pharmacokinetic and pharmacodynamic characterization limits rational dose optimization and patient selection. Future therapeutic development should therefore prioritize the design of brain-penetrant and cell-specific agents, integration with combination therapies (e.g., immunotherapy and radiotherapy), and the identification of predictive biomarkers of ferroptosis sensitivity. Addressing these challenges will be essential to unlock the full therapeutic potential of ferroptosis modulation in both neurodegenerative diseases and CNS malignancies.

### 5.3 Regulation of immune microenvironment

The interaction between ferroptosis, neuroinflammation, and immune cells is involved in the occurrence and development of neuropathological damage or diseases, and neuroinflammation plays a scaffolding role between ferroptosis and immune cells. Numerous studies have shown that after brain injury, inhibiting the massive infiltration of immune cells can effectively reduce neuroinflammation, thereby reducing lipid peroxidation and ferroptosis; conversely, effectively inhibiting lipid peroxidation ferroptosis can reduce neuroinflammation and maintain the homeostasis of the immune microenvironment. Therefore, targeting the interaction between ferroptosis mediated by inflammatory signaling pathways and the immune microenvironment may be a new measure for the clinical treatment of neuropathological injuries or diseases.

Previous studies have elucidated that the Nrf2/NF-κB/GPX4 signaling axis mediates the crosstalk between neuroinflammation and ferroptosis. Therefore, small-molecule drugs targeting this pathway can be used to treat neuropathological injuries or diseases associated with ferroptosis. For instance, icariin II, a natural flavonoid, can block NF-κB-mediated neuroinflammation, oxidative stress, and ferroptosis damage by activating Nrf2 (173). Forsythiaside A treatment protects APP/PS1 double transgenic AD mice from further damage of neuronal ferroptosis and neuroinflammation by activating Nrf2 in microglia, increasing GPX4 expression, and reducing NF-κB activity (174, 175). Saikosaponin B2 alleviates lipid peroxidation ferroptosis by correcting the FTH/GPX4-mediated iron metabolism disorder in hippocampal neurons. At the same time, it also blocks the expression of proinflammatory cytokines mediated by TLR4/NF-κB and inhibits neuroinflammation (176). Quercetin enhances the polarization of M2 microglia and improves brain damage through the PI3K/Akt/NF-κB signaling pathway in I/R. In AD, quercetin can not only effectively reduce the intracellular ROS concentration, but also downregulate the expression of microglial iNOS genes and reduce the expression of proinflammatory cytokines (such as TNF-α, IFN-γ, and IL-6) (177). Additionally, propofol, a conventional anesthetic, fights against gastric cancer, colorectal cancer, and glioma by targeting the JAK/STAT3 pathway to inhibit inflammation, oxidative stress, and ferroptosis (178–180).

Furthermore, targeting immune cells (i.e., CD8^+^ T cells) to block inflammatory responses and ferroptosis is also an ideal strategy to maintain the homeostasis of the immune microenvironment. Previous studies have found that cholesterol-mediated CD8^+^ T cell ferroptosis in the tumor microenvironment leads to decreased anti-tumor immune function. CD36 antibodies block the scavenger receptor CD36 on the surface of T cells, which can prevent T cells from taking up a large amount of unsaturated fatty acids, reduce the occurrence of T cell lipid peroxidation ferroptosis, and thus reduce the secretion of cytotoxic factors (146). Overall, revealing the interaction mechanism between ferroptosis and immune cells provides a theoretical basis for exploring more small molecule drugs for the treatment of neuropathological injuries or diseases.

### 5.4 Combination and nanoparticle-based therapies

The integration of ferroptosis modulators with conventional anti-cancer therapies or immunotherapies represents a promising strategy. Erastin combined with temozolomide enhances SLC7A11-dependent sensitivity in gliomas (181), whereas ferroptosis induction sensitizes tumors to radiotherapy by promoting ROS-mediated lipid peroxidation and ACSL4-dependent PUFA biosynthesis (182). One study reported that combining ferroptosis inhibitors with immune checkpoint blockade (e.g., Fer-1 with anti-PD-L1) reprograms tumor-associated macrophages towards an M1-like phenotype and enhances therapeutic efficacy (141, 183). In neurodegenerative models, co-administration of minocycline (anti-inflammatory) and NAC (ferroptosis inhibitor) ameliorates post-TBI cognitive deficits more effectively than monotherapy (184).

In addition, the application of nanoparticles to precisely regulate cellular ferroptosis is a recent hot research direction. Compared to small molecule, Nanomaterial-induced ferroptosis is more safety and accurate, offering precise, biocompatible approaches to modulate ferroptosis. Many nanomaterials have now been designed to promote ferroptosis by various mechanisms, such as facilitating Fenton reactions and inhibiting GPX4. SRF@FeIIITA nanoparticles combine Fe^3+^, tannic acid, and sorafenib to induce ferroptosis through Fenton reactions and GPX4 inhibition (185). Iron oxide nanoparticles (IONPs) loaded with cisplatin and GPX4 siRNA enhance intracellular ROS while silencing GPX4, providing synergistic anti-glioblastoma effects (186). IONP@PTX triggers ferroptosis via Beclin1-HDAC6-mediated autophagy (187). Advanced multifunctional NMs, including dEGCG-based platforms combined with bispecific antibodies, augment T cell infiltration, IFN-γ release, and tumor ferroptosis (188). There are also related studies on the development of nanomaterials for neurological diseases other than brain tumors. ALNP-DA nanoparticles deliver dopamine across the BBB in PD models, and recombinant human ferritin nanoparticles alleviate oxidative damage and ferroptosis after ischemic stroke (189–191). While these approaches show promise in preclinical models, clinical translation faces substantial hurdles: most nanoparticles achieve only modest brain accumulation with heterogeneous parenchymal distribution, and their long-term safety, biodistribution, and immunogenicity remain poorly characterized (192).

The strategic integration of ferroptosis modulation with immunotherapy and nanotechnology offers a promising therapeutic paradigm for CNS disorders. However, its clinical translation remains constrained by several fundamental challenges. First, limited BBB permeability represents a major bottleneck. Many canonical ferroptosis modulators, including Fer-1 and Lip-1, exhibit suboptimal brain penetration due to unfavorable physicochemical properties. Moreover, in pathological contexts such as glioma or acute brain injury, regional BBB disruption is often heterogeneous, resulting in uneven drug distribution and inconsistent target engagement. These limitations highlight the need for next-generation agents engineered for enhanced CNS delivery, including prodrug strategies, receptor-mediated transcytosis and nanocarrier-based transport systems. Second, off-target effects and safety concerns remain significant obstacles. Because ferroptosis is a fundamental metabolic process, its systemic modulation may inadvertently affect non-target tissues, particularly for broadly acting agents such as iron chelators or GPX4 inhibitors. Achieving cell-type specificity is therefore critical. Emerging strategies, including nanoparticle-based delivery systems and cell-specific prodrugs, offer the potential to preferentially target diseased regions while minimizing systemic toxicity. In parallel, the identification of predictive biomarkers of ferroptosis susceptibility will be essential to refine patient stratification and optimize therapeutic windows. Third, pharmacokinetic and pharmacodynamic limitations of ferroptosis modulators remain insufficiently characterized. Key parameters, including *in vivo* metabolism, protein binding, tissue distribution and drug stability, are often poorly defined, hindering rational dose optimization and clinical translation. Addressing these gaps will require systematic preclinical evaluation of absorption, distribution, metabolism and excretion profiles, alongside the development of formulations that improve bioavailability and enable controlled or sustained release.

Overall, the convergence of ferroptosis-targeted pharmacology, immune modulation, and nanotechnology holds promise for both neurodegenerative and oncological applications. Future studies are required to delineate the precise molecular mechanisms by which ferroptosis modulates anti-tumor and neuroprotective responses, and to optimize combination therapies for maximal clinical benefit.

## 6 Concluding remarks and future directions

Ferroptosis has emerged as a central determinant of cell fate in the CNS, operating at the intersection of metabolic stress, redox imbalance, and immune regulation. Accumulating evidence indicates that dysregulated iron homeostasis, excessive ROS generation, and lipid peroxidation not only drive ferroptotic death in neurons and glial cells, but also actively shape the activation states, differentiation trajectories, and effector functions of both resident and peripheral immune populations. This bidirectional crosstalk establishes self-amplifying feedback loops that exacerbate neurodegeneration, promote demyelination, and remodel the tumor microenvironment, thereby positioning ferroptosis as both a mechanistic driver and a disease amplifier across diverse CNS pathologies.

From a therapeutic perspective, targeting ferroptosis and its interaction with immune cells offers a versatile and context-dependent strategy. In neurodegenerative and inflammatory conditions, ferroptosis inhibitors have shown promise in attenuating neuronal injury and dampening neuroinflammation. In contrast, in CNS malignancies such as glioma, ferroptosis induction represents a potential vulnerability that can be exploited to overcome therapeutic resistance and enhance anti-tumor immunity, particularly when integrated with chemotherapy, radiotherapy, immune checkpoint blockade, or nanomaterial-based delivery systems. Notably, emerging evidence suggests that modulating ferroptosis susceptibility in immune cells themselves may further refine the immune microenvironment and improve therapeutic outcomes.

Despite these advances, several critical gaps remain. The molecular circuitry linking ferroptosis to specific immune cell phenotypes and cytokine networks is incompletely defined, and current models often fail to capture the context-dependent and cell-type-specific nature of these interactions. In particular, the spatiotemporal dynamics of ferroptosis across heterogeneous CNS cell populations and within distinct pathological niches, including inflamed parenchyma and tumor microenvironments, remain poorly resolved. In addition, although nanotechnology-enabled delivery systems and combinatorial therapeutic strategies offer enhanced specificity, their long-term safety, biodistribution, immunogenicity, and regulatory feasibility require careful and systematic evaluation before clinical translation.

Addressing these challenges will require an integrative and multidisciplinary approach. Accordingly, future research should prioritize the following directions: i) Single-cell and multi-omics approaches for mapping cell-type-specific ferroptosis susceptibility and immune interactions. ii) Biomarker development for ferroptosis-immune activity in patients. iii) Ethical and safety considerations in ferroptosis-targeted nanotherapies, including long-term biodistribution, immunogenicity, and appropriate regulatory frameworks. iv) Integration of AI-driven drug discovery for ferroptosis modulators.

In summary, the convergence of ferroptosis biology and immune regulation defines a rapidly evolving frontier in neuroscience and neuro-oncology. A deeper mechanistic understanding, coupled with the development of precise and translationally viable therapeutic strategies, holds the potential to simultaneously mitigate neurodegeneration, promote neural repair, and enhance anti-tumor immunity, ultimately transforming the clinical management of CNS disorders and brain malignancies.

## References

[1] Zhang Q Jia M Wang Y Wang Q Wu J . Cell death mechanisms in cerebral ischemia-reperfusion injury. Neurochem Res. (2022) 47:3525–42. doi: 10.1007/s11064-022-03697-8. PMID: 35976487

[2] Moujalled D Strasser A Liddell JR . Molecular mechanisms of cell death in neurological diseases. Cell Death Differ. (2021) 28:2029–44. doi: 10.1038/s41418-021-00814-y. PMID: 34099897 PMC8257776

[3] Shi Z Yuan S Shi L Li J Ning G Kong X . Programmed cell death in spinal cord injury pathogenesis and therapy. Cell Prolif. (2021) 54:e12992. doi: 10.1111/cpr.12992. PMID: 33506613 PMC7941236

[4] Mahoney-Sánchez L Bouchaoui H Ayton S Devos D Duce JA Devedjian JC . Ferroptosis and its potential role in the physiopathology of Parkinson's disease. Prog Neurobiol. (2021) 196:101890. doi: 10.1016/j.pneurobio.2020.101890. PMID: 32726602

[5] Yan HF Zou T Tuo QZ Xu S Li H Belaidi AA . Ferroptosis: mechanisms and links with diseases. Signal Transduct Target Ther. (2021) 6:49. doi: 10.1038/s41392-020-00428-9. PMID: 33536413 PMC7858612

[6] Li J Cao F Yin HL Huang ZJ Lin ZT Mao N . Ferroptosis: past, present and future. Cell Death Dis. (2020) 11:88. doi: 10.1038/s41419-020-2298-2. PMID: 32015325 PMC6997353

[7] Ko CJ Gao SL Lin TK Chu PY Lin HY . Ferroptosis as a major factor and therapeutic target for neuroinflammation in Parkinson's disease. Biomedicines. (2021) 9(11):1679. doi: 10.3390/biomedicines9111679. PMID: 34829907 PMC8615560

[8] Xu Y Jia B Li J Li Q Luo C . The interplay between ferroptosis and neuroinflammation in central neurological disorders. Antioxidants (Basel). (2024) 13(4):395. doi: 10.3390/antiox13040395. PMID: 38671843 PMC11047682

[9] Mohan S Alhazmi HA Hassani R Khuwaja G Maheshkumar VP Aldahish A . Role of ferroptosis pathways in neuroinflammation and neurological disorders: From pathogenesis to treatment. Heliyon. (2024) 10:e24786. doi: 10.1016/j.heliyon.2024.e24786. PMID: 38314277 PMC10837572

[10] Lee J Hyun DH . The interplay between intracellular iron homeostasis and neuroinflammation in neurodegenerative diseases. Antioxidants (Basel). (2023) 12(4):918. doi: 10.3390/antiox12040918. PMID: 37107292 PMC10135822

[11] Fang D Guo S Wei B Liu W Li G Li X . Nrf-2 modulates excitability of hippocampal neurons by regulating ferroptosis and neuroinflammation after subarachnoid hemorrhage in rats. Brain Res Bull. (2024) 207:110877. doi: 10.1016/j.brainresbull.2024.110877. PMID: 38215951

[12] Wang M Tang G Zhou C Guo H Hu Z Hu Q . Revisiting the intersection of microglial activation and neuroinflammation in Alzheimer's disease from the perspective of ferroptosis. Chem Biol Interact. (2023) 375:110387. doi: 10.1016/j.cbi.2023.110387. PMID: 36758888

[13] Yu H Chang Q Sun T He X Wen L An J . Metabolic reprogramming and polarization of microglia in Parkinson's disease: Role of inflammasome and iron. Ageing Res Rev. (2023) 90:102032. doi: 10.1016/j.arr.2023.102032. PMID: 37572760

[14] Chen LL Fan YG Zhao LX Zhang Q Wang ZY . The metal ion hypothesis of Alzheimer's disease and the anti-neuroinflammatory effect of metal chelators. Bioorg Chem. (2023) 131:106301. doi: 10.1016/j.bioorg.2022.106301. PMID: 36455485

[15] Zhao J Wang Y Tao L Chen L . Iron transporters and ferroptosis in Malignant brain tumors. Front Oncol. (2022) 12:861834. doi: 10.3389/fonc.2022.861834. PMID: 35530363 PMC9071296

[16] Vogt AS Arsiwala T Mohsen M Vogel M Manolova V Bachmann MF . On iron metabolism and its regulation. Int J Mol Sci. (2021) 22(9):4591. doi: 10.3390/ijms22094591. PMID: 33925597 PMC8123811

[17] Costa I Barbosa DJ Benfeito S Silva V Chavarria D Borges F . Molecular mechanisms of ferroptosis and their involvement in brain diseases. Pharmacol Ther. (2023) 244:108373. doi: 10.1016/j.pharmthera.2023.108373. PMID: 36894028

[18] Agmon E Solon J Bassereau P Stockwell BR . Modeling the effects of lipid peroxidation during ferroptosis on membrane properties. Sci Rep. (2018) 8:5155. doi: 10.1038/s41598-018-23408-0. PMID: 29581451 PMC5979948

[19] Song S Su Z Kon N Chu B Li H Jiang X . ALOX5-mediated ferroptosis acts as a distinct cell death pathway upon oxidative stress in Huntington's disease. Genes Dev. (2023) 37:204–17. doi: 10.1101/gad.350211.122. PMID: 36921996 PMC10111862

[20] Ursini F Maiorino M . Lipid peroxidation and ferroptosis: The role of GSH and GPx4. Free Radic Biol Med. (2020) 152:175–85. doi: 10.1016/j.freeradbiomed.2020.02.027. PMID: 32165281

[21] Bersuker K Hendricks JM Li Z Magtanong L Ford B Tang PH . The CoQ oxidoreductase FSP1 acts parallel to GPX4 to inhibit ferroptosis. Nature. (2019) 575:688–92. doi: 10.1038/s41586-019-1705-2. PMID: 31634900 PMC6883167

[22] Kraft VAN Bezjian CT Pfeiffer S Ringelstetter L Müller C Zandkarimi F . GTP cyclohydrolase 1/tetrahydrobiopterin counteract ferroptosis through lipid remodeling. ACS Cent Sci. (2020) 6:41–53. doi: 10.1021/acscentsci.9b01063. PMID: 31989025 PMC6978838

[23] Lv B Wang Y Ma D Cheng W Liu J Yong T . Immunotherapy: Reshape the tumor immune microenvironment. Front Immunol. (2022) 13:844142. doi: 10.3389/fimmu.2022.844142. PMID: 35874717 PMC9299092

[24] Capece D Verzella D Flati I Arboretto P Cornice J Franzoso G . NF-κB: blending metabolism, immunity, and inflammation. Trends Immunol. (2022) 43:757–75. doi: 10.1016/j.it.2022.07.004. PMID: 35965153

[25] Yan N Xu Z Qu C Zhang J . Dimethyl fumarate improves cognitive deficits in chronic cerebral hypoperfusion rats by alleviating inflammation, oxidative stress, and ferroptosis via NRF2/ARE/NF-κB signal pathway. Int Immunopharmacol. (2021) 98:107844. doi: 10.1016/j.intimp.2021.107844. PMID: 34153667

[26] Chen Y Fang ZM Yi X Wei X Jiang DS . The interaction between ferroptosis and inflammatory signaling pathways. Cell Death Dis. (2023) 14:205. doi: 10.1038/s41419-023-05716-0. PMID: 36944609 PMC10030804

[27] Kong R Wang N Han W Bao W Lu J . IFNγ-mediated repression of system xc(-) drives vulnerability to induced ferroptosis in hepatocellular carcinoma cells. J Leukoc Biol. (2021) 110:301–14. doi: 10.1002/jlb.3ma1220-815rrr. PMID: 34318944

[28] Li C Liu J Hou W Kang R Tang D . STING1 promotes ferroptosis through MFN1/2-dependent mitochondrial fusion. Front Cell Dev Biol. (2021) 9:698679. doi: 10.3389/fcell.2021.698679. PMID: 34195205 PMC8236825

[29] Wen Q Liu J Kang R Zhou B Tang D . The release and activity of HMGB1 in ferroptosis. Biochem Biophys Res Commun. (2019) 510:278–83. doi: 10.1016/j.bbrc.2019.01.090. PMID: 30686534

[30] Xu H Ye D Ren M Zhang H Bi F . Ferroptosis in the tumor microenvironment: perspectives for immunotherapy. Trends Mol Med. (2021) 27:856–67. doi: 10.1016/j.molmed.2021.06.014. PMID: 34312075

[31] Li H Edin ML Gruzdev A Cheng J Bradbury JA Graves JP . Regulation of T helper cell subsets by cyclooxygenases and their metabolites. Prostaglandins Other Lipid Mediat. (2013) 104-105:74–83. doi: 10.1016/j.prostaglandins.2012.11.002. PMID: 23201570 PMC3620713

[32] Liu S Gao X Zhou S . New target for prevention and treatment of neuroinflammation: Microglia iron accumulation and ferroptosis. ASN Neuro. (2022) 14:17590914221133236. doi: 10.1177/17590914221133236. PMID: 36285433 PMC9607999

[33] Liu Z Shen X Li M Liu P Ge Z Jin J . Exploring the nexus: How ferroptosis, microglia, and neuroinflammation converge in ischemic stroke pathogenesis. Mol Neurobiol. (2025) 62:8965–76. doi: 10.1007/s12035-025-04815-7. PMID: 40063316

[34] Devos D Cabantchik ZI Moreau C Danel V Mahoney-Sanchez L Bouchaoui H . Conservative iron chelation for neurodegenerative diseases such as Parkinson's disease and amyotrophic lateral sclerosis. J Neural Transm (Vienna). (2020) 127:189–203. doi: 10.1007/s00702-019-02138-1. PMID: 31912279

[35] Cui Y Zhang Z Zhou X Zhao Z Zhao R Xu X . Microglia and macrophage exhibit attenuated inflammatory response and ferroptosis resistance after RSL3 stimulation via increasing Nrf2 expression. J Neuroinflamm. (2021) 18:249. doi: 10.1186/s12974-021-02231-x. PMID: 34717678 PMC8557003

[36] Liang D Minikes AM Jiang X . Ferroptosis at the intersection of lipid metabolism and cellular signaling. Mol Cell. (2022) 82:2215–27. doi: 10.1016/j.molcel.2022.03.022. PMID: 35390277 PMC9233073

[37] Chausse B Kakimoto PA Kann O . Microglia and lipids: how metabolism controls brain innate immunity. Semin Cell Dev Biol. (2021) 112:137–44. doi: 10.1016/j.semcdb.2020.08.001. PMID: 32807643

[38] Dodson M Castro-Portuguez R Zhang DD . NRF2 plays a critical role in mitigating lipid peroxidation and ferroptosis. Redox Biol. (2019) 23:101107. doi: 10.1016/j.redox.2019.101107. PMID: 30692038 PMC6859567

[39] Kapralov AA Yang Q Dar HH Tyurina YY Anthonymuthu TS Kim R . Redox lipid reprogramming commands susceptibility of macrophages and microglia to ferroptotic death. Nat Chem Biol. (2020) 16:278–90. doi: 10.1038/s41589-019-0462-8. PMID: 32080625 PMC7233108

[40] Chou ML Babamale AO Walker TL Cognasse F Blum D Burnouf T . Blood-brain crosstalk: the roles of neutrophils, platelets, and neutrophil extracellular traps in neuropathologies. Trends Neurosci. (2023) 46:764–79. doi: 10.1016/j.tins.2023.06.005. PMID: 37500363

[41] Kim SW Lee H Lee HK Kim ID Lee JK . Neutrophil extracellular trap induced by HMGB1 exacerbates damages in the ischemic brain. Acta Neuropathol Commun. (2019) 7:94. doi: 10.1186/s40478-019-0747-x. PMID: 31177989 PMC6556959

[42] Yee PP Wei Y Kim SY Lu T Chih SY Lawson C . Neutrophil-induced ferroptosis promotes tumor necrosis in glioblastoma progression. Nat Commun. (2020) 11:5424. doi: 10.1038/s41467-020-19193-y. PMID: 33110073 PMC7591536

[43] Zhu Y Xu J Chai Y Li P Liu L Zhang S . Neutrophil extracellular traps aggravate blood-brain barrier disruption via ZBP1/FSP1-mediated ferroptosis after traumatic brain injury. Fluids Barriers CNS. (2025) 23:2. doi: 10.1186/s12987-025-00739-5. PMID: 41331823 PMC12777371

[44] Gong Z Guo J Liu B Guo Y Cheng C Jiang Y . Mechanisms of immune response and cell death in ischemic stroke and their regulation by natural compounds. Front Immunol. (2023) 14:1287857. doi: 10.3389/fimmu.2023.1287857. PMID: 38274789 PMC10808662

[45] Brait VH Arumugam TV Drummond GR Sobey CG . Importance of T lymphocytes in brain injury, immunodeficiency, and recovery after cerebral ischemia. J Cereb Blood Flow Metab. (2012) 32:598–611. doi: 10.1038/jcbfm.2012.6. PMID: 22293986 PMC3318155

[46] Gao J Zhang X Liu Y Gu X . Ferroptosis in immune cells: Implications for tumor immunity and cancer therapy. Cytokine Growth Factor Rev. (2025) 84:59–73. doi: 10.1016/j.cytogfr.2025.06.007. PMID: 40579305

[47] Xu S Chaudhary O Rodríguez-Morales P Sun X Chen D Zappasodi R . Uptake of oxidized lipids by the scavenger receptor CD36 promotes lipid peroxidation and dysfunction in CD8(+) T cells in tumors. Immunity. (2021) 54:1561–77.e7. doi: 10.1016/j.immuni.2021.05.003. PMID: 34102100 PMC9273026

[48] Xu C Sun S Johnson T Qi R Zhang S Zhang J . The glutathione peroxidase Gpx4 prevents lipid peroxidation and ferroptosis to sustain Treg cell activation and suppression of antitumor immunity. Cell Rep. (2021) 35:109235. doi: 10.1016/j.celrep.2021.109235. PMID: 34133924

[49] Li L Xia Y Yuan S Li F Xie X Luo Y . Iron deprivation restrains the differentiation and pathogenicity of T helper 17 cell. J Leukoc Biol. (2021) 110:1057–67. doi: 10.1002/jlb.3ma0821-015r. PMID: 34612525

[50] Colonna M Butovsky O . Microglia function in the central nervous system during health and neurodegeneration. Annu Rev Immunol. (2017) 35:441–68. doi: 10.1146/annurev-immunol-051116-052358. PMID: 28226226 PMC8167938

[51] Kwon HS Koh SH . Neuroinflammation in neurodegenerative disorders: the roles of microglia and astrocytes. Transl Neurodegener. (2020) 9:42. doi: 10.1186/s40035-020-00221-2. PMID: 33239064 PMC7689983

[52] Wang C Zong S Cui X Wang X Wu S Wang L . The effects of microglia-associated neuroinflammation on Alzheimer's disease. Front Immunol. (2023) 14:1117172. doi: 10.3389/fimmu.2023.1117172. PMID: 36911732 PMC9992739

[53] Han RT Kim RD Molofsky AV Liddelow SA . Astrocyte-immune cell interactions in physiology and pathology. Immunity. (2021) 54:211–24. doi: 10.1016/j.immuni.2021.01.013. PMID: 33567261

[54] Singh D . Astrocytic and microglial cells as the modulators of neuroinflammation in Alzheimer's disease. J Neuroinflamm. (2022) 19:206. doi: 10.1186/s12974-022-02565-0. PMID: 35978311 PMC9382837

[55] Lee HG Wheeler MA Quintana FJ . Function and therapeutic value of astrocytes in neurological diseases. Nat Rev Drug Discov. (2022) 21:339–58. doi: 10.1038/s41573-022-00390-x. PMID: 35173313 PMC9081171

[56] Balog BM Sonti A Zigmond RE . Neutrophil biology in injuries and diseases of the central and peripheral nervous systems. Prog Neurobiol. (2023) 228:102488. doi: 10.1016/j.pneurobio.2023.102488. PMID: 37355220 PMC10528432

[57] Li C Xing Y Zhang Y Hua Y Hu J Bai Y . Neutrophil extracellular traps exacerbate ischemic brain damage. Mol Neurobiol. (2022) 59:643–56. doi: 10.1007/s12035-021-02635-z. PMID: 34748205

[58] DeMaio A Mehrotra S Sambamurti K Husain S . The role of the adaptive immune system and T cell dysfunction in neurodegenerative diseases. J Neuroinflamm. (2022) 19:251. doi: 10.1186/s12974-022-02605-9. PMID: 36209107 PMC9548183

[59] Terrabuio E Zenaro E Constantin G . The role of the CD8+ T cell compartment in ageing and neurodegenerative disorders. Front Immunol. (2023) 14:1233870. doi: 10.3389/fimmu.2023.1233870. PMID: 37575227 PMC10416633

[60] Li S Huang P Lai F Zhang T Guan J Wan H . Mechanisms of ferritinophagy and ferroptosis in diseases. Mol Neurobiol. (2024) 61:1605–26. doi: 10.1007/s12035-023-03640-0. PMID: 37736794

[61] Xu S He Y Lin L Chen P Chen M Zhang S . The emerging role of ferroptosis in intestinal disease. Cell Death Dis. (2021) 12:289. doi: 10.1038/s41419-021-03559-1. PMID: 33731703 PMC7969743

[62] Li J Jia B Cheng Y Song Y Li Q Luo C . Targeting molecular mediators of ferroptosis and oxidative stress for neurological disorders. Oxid Med Cell Longev. (2022) 2022:3999083. doi: 10.1155/2022/3999083. PMID: 35910843 PMC9337979

[63] Huang X . A concise review on oxidative stress-mediated ferroptosis and cuproptosis in alzheimer's disease. Cells. (2023) 12(10):1369. doi: 10.3390/cells12101369. PMID: 37408203 PMC10216514

[64] Qiu Z Zhang H Xia M Gu J Guo K Wang H . Programmed death of microglia in alzheimer's disease: autophagy, ferroptosis, and pyroptosis. J Prev Alzheimers Dis. (2023) 10:95–103. doi: 10.14283/jpad.2023.3. PMID: 36641613

[65] Zeineh MM Chen Y Kitzler HH Hammond R Vogel H Rutt BK . Activated iron-containing microglia in the human hippocampus identified by magnetic resonance imaging in Alzheimer disease. Neurobiol Aging. (2015) 36:2483–500. doi: 10.1016/j.neurobiolaging.2015.05.022. PMID: 26190634 PMC4839298

[66] Kroner A Greenhalgh AD Zarruk JG Passos Dos Santos R Gaestel M David S . TNF and increased intracellular iron alter macrophage polarization to a detrimental M1 phenotype in the injured spinal cord. Neuron. (2014) 83:1098–116. doi: 10.1016/j.neuron.2014.07.027. PMID: 25132469

[67] McCarthy RC Sosa JC Gardeck AM Baez AS Lee CH Wessling-Resnick M . Inflammation-induced iron transport and metabolism by brain microglia. J Biol Chem. (2018) 293:7853–63. doi: 10.1074/jbc.ra118.001949. PMID: 29610275 PMC5961037

[68] Wang J Song N Jiang H Wang J Xie J . Pro-inflammatory cytokines modulate iron regulatory protein 1 expression and iron transportation through reactive oxygen/nitrogen species production in ventral mesencephalic neurons. Biochim Biophys Acta. (2013) 1832:618–25. doi: 10.1016/j.bbadis.2013.01.021. PMID: 23376588

[69] Urrutia P Aguirre P Esparza A Tapia V Mena NP Arredondo M . Inflammation alters the expression of DMT1, FPN1 and hepcidin, and it causes iron accumulation in central nervous system cells. J Neurochem. (2013) 126:541–9. doi: 10.1111/jnc.12244. PMID: 23506423

[70] Cheng Y Song Y Chen H Li Q Gao Y Lu G . Ferroptosis mediated by lipid reactive oxygen species: A possible causal link of neuroinflammation to neurological disorders. Oxid Med Cell Longev. (2021) 2021:5005136. doi: 10.1155/2021/5005136. PMID: 34725564 PMC8557075

[71] Hashioka S Wu Z Klegeris A . Glia-driven neuroinflammation and systemic inflammation in alzheimer's disease. Curr Neuropharmacol. (2021) 19:908–24. doi: 10.2174/1570159x18666201111104509. PMID: 33176652 PMC8686312

[72] Ding XS Gao L Han Z Eleuteri S Shi W Shen Y . Ferroptosis in Parkinson's disease: Molecular mechanisms and therapeutic potential. Ageing Res Rev. (2023) 91:102077. doi: 10.1016/j.arr.2023.102077. PMID: 37742785

[73] Park MW Cha HW Kim J Kim JH Yang H Yoon S . NOX4 promotes ferroptosis of astrocytes by oxidative stress-induced lipid peroxidation via the impairment of mitochondrial metabolism in Alzheimer's diseases. Redox Biol. (2021) 41:101947. doi: 10.1016/j.redox.2021.101947. PMID: 33774476 PMC8027773

[74] Wang F Wang J Shen Y Li H Rausch WD Huang X . Iron dyshomeostasis and ferroptosis: A new alzheimer's disease hypothesis? Front Aging Neurosci. (2022) 14:830569. doi: 10.3389/fnagi.2022.830569. PMID: 35391749 PMC8981915

[75] Liu Q Song T Chen B Zhang J Li W . Ferroptosis of brain microvascular endothelial cells contributes to hypoxia-induced blood-brain barrier injury. FASEB J. (2023) 37:e22874. doi: 10.1096/fj.202201765r. PMID: 37043308

[76] Li J Li M Ge Y Chen J Ma J Wang C . β-amyloid protein induces mitophagy-dependent ferroptosis through the CD36/PINK/PARKIN pathway leading to blood-brain barrier destruction in Alzheimer's disease. Cell Biosci. (2022) 12:69. doi: 10.21203/rs.3.rs-1087555/v1. PMID: 35619150 PMC9134700

[77] Gao Y Lu Y Liang X Zhao M Yu X Fu H . CD4(+) T-cell senescence in neurodegenerative disease: pathogenesis and potential therapeutic targets. Cells. (2024) 13(9):749. doi: 10.3390/cells13090749. PMID: 38727285 PMC11083511

[78] Zhang J Ke KF Liu Z Qiu YH Peng YP . Th17 cell-mediated neuroinflammation is involved in neurodegeneration of aβ1-42-induced Alzheimer's disease model rats. PloS One. (2013) 8:e75786. doi: 10.1371/journal.pone.0075786. PMID: 24124514 PMC3790825

[79] Hu D Weiner HL . Unraveling the dual nature of brain CD8(+) T cells in Alzheimer's disease. Mol Neurodegener. (2024) 19:16. doi: 10.1186/s13024-024-00706-y. PMID: 38355649 PMC10865558

[80] Elsworth JD . Parkinson's disease treatment: past, present, and future. J Neural Transm (Vienna). (2020) 127:785–91. doi: 10.1007/s00702-020-02167-1. PMID: 32172471 PMC8330829

[81] Morris HR Spillantini MG Sue CM Williams-Gray CH . The pathogenesis of Parkinson's disease. Lancet. (2024) 403:293–304. doi: 10.1016/s0889-8529(05)70299-1. PMID: 38245249

[82] Jankovic J Tan EK . Parkinson's disease: etiopathogenesis and treatment. J Neurol Neurosurg Psychiatry. (2020) 91:795–808. doi: 10.1136/jnnp-2019-322338. PMID: 32576618

[83] Wang Q Liu J Zhang Y Li Z Zhao Z Jiang W . Microglial CR3 promotes neuron ferroptosis via NOX2-mediated iron deposition in rotenone-induced experimental models of Parkinson's disease. Redox Biol. (2024) 77:103369. doi: 10.1016/j.redox.2024.103369. PMID: 39357423 PMC11471230

[84] Zhang B Chen K Dai Y Luo X Xiong Z Zhang W . Human α-synuclein aggregation activates ferroptosis leading to parvalbumin interneuron degeneration and motor learning impairment. Commun Biol. (2024) 7:1227. doi: 10.1038/s42003-024-06896-x. PMID: 39349708 PMC11443099

[85] Wang ZL Yuan L Li W Li JY . Ferroptosis in Parkinson's disease: glia-neuron crosstalk. Trends Mol Med. (2022) 28:258–69. doi: 10.1016/j.molmed.2022.02.003. PMID: 35260343

[86] Kam TI Hinkle JT Dawson TM Dawson VL . Microglia and astrocyte dysfunction in parkinson's disease. Neurobiol Dis. (2020) 144:105028. doi: 10.1016/j.nbd.2020.105028. PMID: 32736085 PMC7484088

[87] Song N Wang J Jiang H Xie J . Astroglial and microglial contributions to iron metabolism disturbance in Parkinson's disease. Biochim Biophys Acta Mol Basis Dis. (2018) 1864:967–73. doi: 10.1016/j.bbadis.2018.01.008. PMID: 29317336

[88] Cheli VT Correale J Paez PM Pasquini JM . Iron metabolism in oligodendrocytes and astrocytes, implications for myelination and remyelination. ASN Neuro. (2020) 12:1759091420962681. doi: 10.1177/1759091420962681. PMID: 32993319 PMC7545512

[89] Shih AY Johnson DA Wong G Kraft AD Jiang L Erb H . Coordinate regulation of glutathione biosynthesis and release by nrf2-expressing glia potently protects neurons from oxidative stress. J Neurosci. (2003) 23:3394–406. doi: 10.1523/jneurosci.23-08-03394.2003. PMID: 12716947 PMC6742304

[90] Khor SLQ Ng KY Koh RY Chye SM . Blood-brain barrier and neurovascular unit dysfunction in parkinson's disease: from clinical insights to pathogenic mechanisms and novel therapeutic approaches. CNS Neurol Disord Drug Targets. (2024) 23:315–30. doi: 10.2174/1871527322666230330093829. PMID: 36999187

[91] Pajares M A IR Manda G Boscá L Cuadrado A . Inflammation in parkinson's disease: mechanisms and therapeutic implications. Cells. (2020) 9(7):1687. doi: 10.3390/cells9071687. PMID: 32674367 PMC7408280

[92] Williams GP Schonhoff AM Jurkuvenaite A Gallups NJ Standaert DG Harms AS . CD4 T cells mediate brain inflammation and neurodegeneration in a mouse model of Parkinson's disease. Brain. (2021) 144:2047–59. doi: 10.1093/brain/awab103. PMID: 33704423 PMC8370411

[93] Baba Y Kuroiwa A Uitti RJ Wszolek ZK Yamada T . Alterations of T-lymphocyte populations in Parkinson disease. Parkinsonism Relat Disord. (2005) 11:493–8. doi: 10.1016/j.parkreldis.2005.07.005. PMID: 16154792

[94] Schonhoff AM Williams GP Wallen ZD Standaert DG Harms AS . Innate and adaptive immune responses in Parkinson's disease. Prog Brain Res. (2020) 252:169–216. doi: 10.1016/bs.pbr.2019.10.006, PMID: 32247364 PMC7185735

[95] Harms AS Cao S Rowse AL Thome AD Li X Mangieri LR . MHCII is required for α-synuclein-induced activation of microglia, CD4 T cell proliferation, and dopaminergic neurodegeneration. J Neurosci. (2013) 33:9592–600. doi: 10.1523/jneurosci.5610-12.2013. PMID: 23739956 PMC3903980

[96] Iba M Kim C Sallin M Kwon S Verma A Overk C . Neuroinflammation is associated with infiltration of T cells in Lewy body disease and α-synuclein transgenic models. J Neuroinflamm. (2020) 17:214. doi: 10.1186/s12974-020-01888-0. PMID: 32680537 PMC7368752

[97] Mead RJ Shan N Reiser HJ Marshall F Shaw PJ . Amyotrophic lateral sclerosis: a neurodegenerative disorder poised for successful therapeutic translation. Nat Rev Drug Discov. (2023) 22:185–212. doi: 10.1038/s41573-022-00612-2. PMID: 36543887 PMC9768794

[98] Yang B Pan J Zhang XN Wang H He L Rong X . NRF2 activation suppresses motor neuron ferroptosis induced by the SOD1(G93A) mutation and exerts neuroprotection in amyotrophic lateral sclerosis. Neurobiol Dis. (2023) 184:106210. doi: 10.1016/j.nbd.2023.106210. PMID: 37352984

[99] Tu LF Zhang TZ Zhou YF Zhou QQ Gong HB Liang L . GPX4 deficiency-dependent phospholipid peroxidation drives motor deficits of ALS. J Adv Res. (2023) 43:205–18. doi: 10.1016/j.jare.2022.02.016. PMID: 36585109 PMC9811330

[100] Lu CH Allen K Oei F Leoni E Kuhle J Tree T . Systemic inflammatory response and neuromuscular involvement in amyotrophic lateral sclerosis. Neurol Neuroimmunol Neuroinflamm. (2016) 3:e244. doi: 10.1212/nxi.0000000000000244. PMID: 27308305 PMC4897985

[101] Calafatti M Cocozza G Limatola C Garofalo S . Microglial crosstalk with astrocytes and immune cells in amyotrophic lateral sclerosis. Front Immunol. (2023) 14:1223096. doi: 10.3389/fimmu.2023.1223096. PMID: 37564648 PMC10410456

[102] Li Z Zhang Y Ji M Wu C Zhang Y Ji S . Targeting ferroptosis in neuroimmune and neurodegenerative disorders for the development of novel therapeutics. BioMed Pharmacother. (2024) 176:116777. doi: 10.1016/j.biopha.2024.116777. PMID: 38795640

[103] Liddell JR Hilton JBW Kysenius K Billings JL Nikseresht S McInnes LE . Microglial ferroptotic stress causes non-cell autonomous neuronal death. Mol Neurodegener. (2024) 19:14. doi: 10.1186/s13024-023-00691-8. PMID: 38317225 PMC10840184

[104] Ingelfinger F De Feo D Becher B . GM-CSF: Master regulator of the T cell-phagocyte interface during inflammation. Semin Immunol. (2021) 54:101518. doi: 10.1016/j.smim.2021.101518. PMID: 34763973

[105] Siotto M Filippi MM Simonelli I Landi D Ghazaryan A Vollaro S . Oxidative stress related to iron metabolism in relapsing remitting multiple sclerosis patients with low disability. Front Neurosci. (2019) 13:86. doi: 10.3389/fnins.2019.00086. PMID: 30804745 PMC6378854

[106] Duarte-Silva E Meuth SG Peixoto CA . The role of iron metabolism in the pathogenesis and treatment of multiple sclerosis. Front Immunol. (2023) 14:1137635. doi: 10.3389/fimmu.2023.1137635. PMID: 37006264 PMC10064139

[107] Wang Z Yin W Zhu L Li J Yao Y Chen F . Iron drives T helper cell pathogenicity by promoting RNA-binding protein PCBP1-mediated proinflammatory cytokine production. Immunity. (2018) 49:80–92.e7. doi: 10.1016/j.immuni.2018.05.008. PMID: 29958803

[108] Luoqian J Yang W Ding X Tuo QZ Xiang Z Zheng Z . Ferroptosis promotes T-cell activation-induced neurodegeneration in multiple sclerosis. Cell Mol Immunol. (2022) 19:913–24. doi: 10.1038/s41423-022-00883-0. PMID: 35676325 PMC9338013

[109] Sriram S Steiner I . Experimental allergic encephalomyelitis: a misleading model of multiple sclerosis. Ann Neurol. (2005) 58:939–45. doi: 10.1002/ana.20743. PMID: 16315280

[110] Jelcic I Al Nimer F Wang J Lentsch V Planas R Jelcic I . Memory B cells activate brain-homing, autoreactive CD4(+) T cells in multiple sclerosis. Cell. (2018) 175:85–100.e23. doi: 10.21417/b7cw5v 30173916 PMC6191934

[111] D'Amico E Zanghì A Gastaldi M Patti F Zappia M Franciotta D . Placing CD20-targeted B cell depletion in multiple sclerosis therapeutic scenario: present and future perspectives. Autoimmun Rev. (2019) 18:665–72. doi: 10.1016/j.autrev.2019.05.003, PMID: 31059839

[112] Hauser SL Waubant E Arnold DL Vollmer T Antel J Fox RJ . B-cell depletion with rituximab in relapsing-remitting multiple sclerosis. N Engl J Med. (2008) 358:676–88. doi: 10.1056/nejmoa0706383. PMID: 18272891

[113] Qin D Li D Wang C Guo S . Ferroptosis and central nervous system demyelinating diseases. J Neurochem. (2023) 165:759–71. doi: 10.1111/jnc.15831. PMID: 37095635

[114] Li X Chu Y Ma R Dou M Li S Song Y . Ferroptosis as a mechanism of oligodendrocyte loss and demyelination in experimental autoimmune encephalomyelitis. J Neuroimmunol. (2022) 373:577995. doi: 10.1016/j.jneuroim.2022.577995. PMID: 36327618

[115] You LH Yan CZ Zheng BJ Ci YZ Chang SY Yu P . Astrocyte hepcidin is a key factor in LPS-induced neuronal apoptosis. Cell Death Dis. (2017) 8:e2676. doi: 10.1038/cddis.2017.93. PMID: 28300826 PMC5386583

[116] Hametner S Wimmer I Haider L Pfeifenbring S Brück W Lassmann H . Iron and neurodegeneration in the multiple sclerosis brain. Ann Neurol. (2013) 74:848–61. doi: 10.1002/ana.23974. PMID: 23868451 PMC4223935

[117] Núñez MT Urrutia P Mena N Aguirre P Tapia V Salazar J . Iron toxicity in neurodegeneration. Biometals. (2012) 25:761–76. doi: 10.1007/s10534-012-9523-0, PMID: 22318507

[118] Campbell BCV De Silva DA Macleod MR Coutts SB Schwamm LH Davis SM . Ischaemic stroke. Nat Rev Dis Primers. (2019) 5:70. doi: 10.1093/med/9780199641208.003.0009. PMID: 31601801

[119] Barthels D Das H . Current advances in ischemic stroke research and therapies. Biochim Biophys Acta Mol Basis Dis. (2020) 1866:165260. doi: 10.1016/j.bbadis.2018.09.012. PMID: 31699365 PMC6981280

[120] Yu Y Yan Y Niu F Wang Y Chen X Su G . Ferroptosis: a cell death connecting oxidative stress, inflammation and cardiovascular diseases. Cell Death Discov. (2021) 7:193. doi: 10.1038/s41420-021-00579-w. PMID: 34312370 PMC8313570

[121] Guo J Tuo QZ Lei P . Iron, ferroptosis, and ischemic stroke. J Neurochem. (2023) 165:487–520. doi: 10.1111/jnc.15807. PMID: 36908209

[122] Candelario-Jalil E Dijkhuizen RM Magnus T . Neuroinflammation, stroke, blood-brain barrier dysfunction, and imaging modalities. Stroke. (2022) 53:1473–86. doi: 10.1161/strokeaha.122.036946. PMID: 35387495 PMC9038693

[123] She R Liu D Liao J Wang G Ge J Mei Z . Mitochondrial dysfunctions induce PANoptosis and ferroptosis in cerebral ischemia/reperfusion injury: from pathology to therapeutic potential. Front Cell Neurosci. (2023) 17:1191629. doi: 10.3389/fncel.2023.1191629. PMID: 37293623 PMC10244524

[124] Bock FJ Tait SWG . Mitochondria as multifaceted regulators of cell death. Nat Rev Mol Cell Biol. (2020) 21:85–100. doi: 10.1038/s41580-019-0173-8. PMID: 31636403

[125] Li C Sun G Chen B Xu L Ye Y He J . Nuclear receptor coactivator 4-mediated ferritinophagy contributes to cerebral ischemia-induced ferroptosis in ischemic stroke. Pharmacol Res. (2021) 174:105933. doi: 10.1016/j.phrs.2021.105933. PMID: 34634471

[126] Ma X Xin D She R Liu D Ge J Mei Z . Novel insight into cGAS-STING pathway in ischemic stroke: from pre- to post-disease. Front Immunol. (2023) 14:1275408. doi: 10.3389/fimmu.2023.1275408. PMID: 37915571 PMC10616885

[127] Tao L Lemoff A Wang G Zarek C Lowe A Yan N . Reactive oxygen species oxidize STING and suppress interferon production. Elife. (2020) 9:e57837. doi: 10.7554/elife.57837. PMID: 32886065 PMC7473769

[128] Liao J Wei M Wang J Zeng J Liu D Du Q . Naotaifang formula attenuates OGD/R-induced inflammation and ferroptosis by regulating microglial M1/M2 polarization through BMP6/SMADs signaling pathway. BioMed Pharmacother. (2023) 167:115465. doi: 10.1016/j.biopha.2023.115465. PMID: 37713988

[129] García-Pupo L Van San E Delgado-Hernández R Vanden Berghe T Vanden Berghe W . Emerging immune and cell death mechanisms in stroke: saponins as therapeutic candidates. Brain Behav Immun Health. (2020) 9:100152. doi: 10.1016/j.bbih.2020.100152. PMID: 34589895 PMC8474497

[130] Lah TT Novak M Breznik B . Brain Malignancies: glioblastoma and brain metastases. Semin Cancer Biol. (2020) 60:262–73. doi: 10.1016/j.semcancer.2019.10.010. PMID: 31654711

[131] Le Rhun E Preusser M Roth P Reardon DA van den Bent M Wen P . Molecular targeted therapy of glioblastoma. Cancer Treat Rev. (2019) 80:101896. doi: 10.1016/j.ctrv.2019.101896. PMID: 31541850

[132] Chaligne R Gaiti F Silverbush D Schiffman JS Weisman HR Kluegel L . Epigenetic encoding, heritability and plasticity of glioma transcriptional cell states. Nat Genet. (2021) 53:1469–79. doi: 10.1038/s41588-021-00927-7. PMID: 34594037 PMC8675181

[133] Lim M Xia Y Bettegowda C Weller M . Current state of immunotherapy for glioblastoma. Nat Rev Clin Oncol. (2018) 15:422–42. doi: 10.1038/s41571-018-0003-5. PMID: 29643471

[134] Chen X Pang X Yeo AJ Xie S Xiang M Shi B . The molecular mechanisms of ferroptosis and its role in blood-brain barrier dysfunction. Front Cell Neurosci. (2022) 16:889765. doi: 10.3389/fncel.2022.889765. PMID: 35663422 PMC9160190

[135] Wang C Wen L Wang K Wu R Li M Zhang Y . Visualization of ferroptosis in brain diseases and ferroptosis-inducing nanomedicine for glioma. Am J Nucl Med Mol Imaging. (2023) 13:179–94. doi: 10.3724/zdxbyxb-2024-0566. PMID: 38023817 PMC10656630

[136] Luo Y Tian G Fang X Bai S Yuan G Pan Y . Ferroptosis and its potential role in glioma: from molecular mechanisms to therapeutic opportunities. Antioxidants (Basel). (2022) 11(11):2123. doi: 10.3390/antiox11112123. PMID: 36358495 PMC9686959

[137] Klöditz K Fadeel B . Three cell deaths and a funeral: macrophage clearance of cells undergoing distinct modes of cell death. Cell Death Discov. (2019) 5:65. doi: 10.1038/s41420-019-0146-x, PMID: 30774993 PMC6368547

[138] Lowenstein PR Castro MG . The long and winding road: from the high-affinity choline uptake site to clinical trials for Malignant brain tumors. Adv Pharmacol. (2016) 76:147–73. doi: 10.1016/bs.apha.2016.03.002. PMID: 27288077 PMC4997808

[139] Wang K Wang J Zhang J Zhang A Liu Y Zhou J . Ferroptosis in glioma immune microenvironment: opportunity and challenge. Front Oncol. (2022) 12:917634. doi: 10.3389/fonc.2022.917634. PMID: 35832539 PMC9273259

[140] Chi H Li B Wang Q Gao Z Feng B Xue H . Opportunities and challenges related to ferroptosis in glioma and neuroblastoma. Front Oncol. (2023) 13:1065994. doi: 10.3389/fonc.2023.1065994. PMID: 36937406 PMC10021024

[141] Liu T Zhu C Chen X Guan G Zou C Shen S . Ferroptosis, as the most enriched programmed cell death process in glioma, induces immunosuppression and immunotherapy resistance. Neuro Oncol. (2022) 24:1113–25. doi: 10.1093/neuonc/noac033. PMID: 35148413 PMC9248406

[142] Zhu Y Yao S Chen L . Cell surface signaling molecules in the control of immune responses: a tide model. Immunity. (2011) 34:466–78. doi: 10.1016/j.immuni.2011.04.008. PMID: 21511182 PMC3176719

[143] Guo X Zhao Y Yan H Yang Y Shen S Dai X . Single tumor-initiating cells evade immune clearance by recruiting type II macrophages. Genes Dev. (2017) 31:247–59. doi: 10.1101/gad.294348.116. PMID: 28223311 PMC5358722

[144] Chen X Kang R Kroemer G Tang D . Broadening horizons: the role of ferroptosis in cancer. Nat Rev Clin Oncol. (2021) 18:280–96. doi: 10.1038/s41571-020-00462-0. PMID: 33514910

[145] Drijvers JM Gillis JE Muijlwijk T Nguyen TH Gaudiano EF Harris IS . Pharmacologic screening identifies metabolic vulnerabilities of CD8(+) T cells. Cancer Immunol Res. (2021) 9:184–95. doi: 10.1158/2326-6066.cir-20-0384. PMID: 33277233 PMC7864883

[146] Ma X Xiao L Liu L Ye L Su P Bi E . CD36-mediated ferroptosis dampens intratumoral CD8(+) T cell effector function and impairs their antitumor ability. Cell Metab. (2021) 33:1001–1012.e5. doi: 10.1016/j.cmet.2021.02.015. PMID: 33691090 PMC8102368

[147] Lei G Zhuang L Gan B . Targeting ferroptosis as a vulnerability in cancer. Nat Rev Cancer. (2022) 22:381–96. doi: 10.1038/s41568-022-00459-0. PMID: 35338310 PMC10243716

[148] Liu B Ji Q Cheng Y Liu M Zhang B Mei Q . Biomimetic GBM-targeted drug delivery system boosting ferroptosis for immunotherapy of orthotopic drug-resistant GBM. J Nanobiotechnology. (2022) 20:161. doi: 10.1186/s12951-022-01360-6. PMID: 35351131 PMC8962245

[149] Shi L Liu Y Li M Luo Z . Emerging roles of ferroptosis in the tumor immune landscape: from danger signals to anti-tumor immunity. FEBS J. (2022) 289:3655–65. doi: 10.1111/febs.16034. PMID: 34042258

[150] Hu J Cheng M Jiang C Liu L He Z Liu L . Deferoxamine mitigates ferroptosis and inflammation in hippocampal neurons after subarachnoid hemorrhage by activating the Nrf2/TXNRD1 axis. Mol Neurobiol. (2024) 61:1044–60. doi: 10.1007/s12035-023-03525-2. PMID: 37676391

[151] Farr AC Xiong MP . Challenges and opportunities of deferoxamine delivery for treatment of Alzheimer's disease, Parkinson's disease, and intracerebral hemorrhage. Mol Pharm. (2021) 18:593–609. doi: 10.1021/acs.molpharmaceut.0c00474. PMID: 32926630 PMC8819678

[152] Selim M Foster LD Moy CS Xi G Hill MD Morgenstern LB . Deferoxamine mesylate in patients with intracerebral haemorrhage (i-DEF): a multicentre, randomised, placebo-controlled, double-blind phase 2 trial. Lancet Neurol. (2019) 18:428–38. doi: 10.1016/s1474-4422(19)30069-9. PMID: 30898550 PMC6494117

[153] Rayatpour A Foolad F Heibatollahi M Khajeh K Javan M . Ferroptosis inhibition by deferiprone, attenuates myelin damage and promotes neuroprotection in demyelinated optic nerve. Sci Rep. (2022) 12:19630. doi: 10.1038/s41598-022-24152-2. PMID: 36385152 PMC9668997

[154] Devos D Labreuche J Rascol O Corvol JC Duhamel A Guyon Delannoy P . Trial of deferiprone in Parkinson's disease. N Engl J Med. (2022) 387:2045–55. doi: 10.1056/nejmoa2209254. PMID: 36449420

[155] Lesley JF Schulte RJ . Inhibition of cell growth by monoclonal anti-transferrin receptor antibodies. Mol Cell Biol. (1985) 5:1814–21. doi: 10.1128/mcb.5.8.1814-1821.1985. PMID: 3018527 PMC366896

[156] Brekelmans P van Soest P Leenen PJ van Ewijk W . Inhibition of proliferation and differentiation during early T cell development by anti-transferrin receptor antibody. Eur J Immunol. (1994) 24:2896–902. doi: 10.1002/eji.1830241147. PMID: 7957580

[157] Shi Y Han L Zhang X Xie L Pan P Chen F . Selenium alleviates cerebral ischemia/reperfusion injury by regulating oxidative stress, mitochondrial fusion and ferroptosis. Neurochem Res. (2022) 47:2992–3002. doi: 10.1007/s11064-022-03643-8. PMID: 35725978 PMC9470641

[158] Zhang Y Sun C Zhao C Hao J Zhang Y Fan B . Ferroptosis inhibitor SRS 16–86 attenuates ferroptosis and promotes functional recovery in contusion spinal cord injury. Brain Res. (2019) 1706:48–57. doi: 10.1016/j.brainres.2018.10.023. PMID: 30352209

[159] Fan BY Pang YL Li WX Zhao CX Zhang Y Wang X . Liproxstatin-1 is an effective inhibitor of oligodendrocyte ferroptosis induced by inhibition of glutathione peroxidase 4. Neural Regener Res. (2021) 16:561–6. doi: 10.4103/1673-5374.293157. PMID: 32985488 PMC7996026

[160] Cao Y Li Y He C Yan F Li JR Xu HZ . Selective ferroptosis inhibitor Liproxstatin-1 attenuates neurological deficits and neuroinflammation after subarachnoid hemorrhage. Neurosci Bull. (2021) 37:535–49. doi: 10.1007/s12264-020-00620-5. PMID: 33421025 PMC8055759

[161] Spasić S Nikolić-Kokić A Miletić S Oreščanin-Dušić Z Spasić MB Blagojević D . Edaravone may prevent ferroptosis in ALS. Curr Drug Targets. (2020) 21:776–80. doi: 10.2174/1389450121666200220123305, PMID: 32077821

[162] Iguchi Y Katsuno M . Current status of drug development for amyotrophic lateral sclerosis. Brain Nerve. (2024) 76:1241–9. doi: 10.11477/mf.1416202766, PMID: 39523617

[163] Zhang Y Tan H Daniels JD Zandkarimi F Liu H Brown LM . Imidazole ketone erastin induces ferroptosis and slows tumor growth in a mouse lymphoma model. Cell Chem Biol. (2019) 26:623–633.e9. doi: 10.1016/j.chembiol.2019.01.008. PMID: 30799221 PMC6525071

[164] Bao Z Hua L Ye Y Wang D Li C Xie Q . MEF2C silencing downregulates NF2 and E-cadherin and enhances erastin-induced ferroptosis in meningioma. Neuro Oncol. (2021) 23:2014–27. doi: 10.1093/neuonc/noab114. PMID: 33984142 PMC8643460

[165] Wang X Lu S He C Wang C Wang L Piao M . RSL3 induced autophagic death in glioma cells via causing glycolysis dysfunction. Biochem Biophys Res Commun. (2019) 518:590–7. doi: 10.1016/j.bbrc.2019.08.096. PMID: 31445705

[166] Song H Liang J Guo Y Liu Y Sa K Yan G . A potent GPX4 degrader to induce ferroptosis in HT1080 cells. Eur J Med Chem. (2024) 265:116110. doi: 10.1016/j.ejmech.2023.116110. PMID: 38194774

[167] Shimada K Skouta R Kaplan A Yang WS Hayano M Dixon SJ . Global survey of cell death mechanisms reveals metabolic regulation of ferroptosis. Nat Chem Biol. (2016) 12:497–503. doi: 10.1038/nchembio.2079. PMID: 27159577 PMC4920070

[168] Zhang X Guo Y Li H Han L . FIN56, a novel ferroptosis inducer, triggers lysosomal membrane permeabilization in a TFEB-dependent manner in glioblastoma. J Cancer. (2021) 12:6610–9. doi: 10.7150/jca.58500. PMID: 34659551 PMC8517990

[169] Siegelin MD Raskett CM Gilbert CA Ross AH Altieri DC . Sorafenib exerts anti-glioma activity *in vitro* and *in vivo*. Neurosci Lett. (2010) 478:165–70. doi: 10.1016/j.neulet.2010.05.009. PMID: 20470863 PMC3198851

[170] Li Q Chen K Zhang T Jiang D Chen L Jiang J . Understanding sorafenib-induced ferroptosis and resistance mechanisms: Implications for cancer therapy. Eur J Pharmacol. (2023) 955:175913. doi: 10.1016/j.ejphar.2023.175913. PMID: 37460053

[171] Arneson K Mondschein J Stavas M Cmelak AJ Attia A Horn L . A phase I trial of concurrent sorafenib and stereotactic radiosurgery for patients with brain metastases. J Neuro-Oncol. (2017) 133:435–42. doi: 10.1007/s11060-017-2455-3. PMID: 28488066

[172] Robe PA Martin DH Nguyen-Khac MT Artesi M Deprez M Albert A . Early termination of ISRCTN45828668, a phase 1/2 prospective, randomized study of sulfasalazine for the treatment of progressing Malignant gliomas in adults. BMC Cancer. (2009) 9:372. doi: 10.1186/1471-2407-9-372. PMID: 19840379 PMC2771045

[173] Gao J Ma C Xia D Chen N Zhang J Xu F . Icariside II preconditioning evokes robust neuroprotection against ischaemic stroke, by targeting Nrf2 and the OXPHOS/NF-κB/ferroptosis pathway. Br J Pharmacol. (2023) 180:308–29. doi: 10.1111/bph.15961. PMID: 36166825

[174] Wang HM Wang LW Liu XM Li CL Xu SP Farooq AD . Neuroprotective effects of forsythiaside on learning and memory deficits in senescence-accelerated mouse prone (SAMP8) mice. Pharmacol Biochem Behav. (2013) 105:134–41. doi: 10.1016/j.pbb.2012.12.016. PMID: 23290932

[175] Wang C Chen S Guo H Jiang H Liu H Fu H . Forsythoside A mitigates Alzheimer's-like pathology by inhibiting ferroptosis-mediated neuroinflammation via Nrf2/GPX4 axis activation. Int J Biol Sci. (2022) 18:2075–90. doi: 10.7150/ijbs.69714. PMID: 35342364 PMC8935224

[176] Wang X Li S Yu J Wang W Du Z Gao S . Saikosaponin B2 ameliorates depression-induced microglia activation by inhibiting ferroptosis-mediated neuroinflammation and ER stress. J Ethnopharmacol. (2023) 316:116729. doi: 10.1016/j.jep.2023.116729. PMID: 37277081

[177] Khan H Ullah H Aschner M Cheang WS Akkol EK . Neuroprotective effects of quercetin in Alzheimer's disease. Biomolecules. (2019) 10(1):59. doi: 10.3390/biom10010059. PMID: 31905923 PMC7023116

[178] Zhao X Chen F . Propofol induces the ferroptosis of colorectal cancer cells by downregulating STAT3 expression. Oncol Lett. (2021) 22:767. doi: 10.3892/ol.2021.13028. PMID: 34589146 PMC8442167

[179] Zhang Y Zuo Y Li B Xie J Ma Z Thirupathi A . Propofol prevents oxidative stress and apoptosis by regulating iron homeostasis and targeting JAK/STAT3 signaling in SH-SY5Y cells. Brain Res Bull. (2019) 153:191–201. doi: 10.1016/j.brainresbull.2019.08.018. PMID: 31472185

[180] Liu YP Qiu ZZ Li XH Li EY . Propofol induces ferroptosis and inhibits Malignant phenotypes of gastric cancer cells by regulating miR-125b-5p/STAT3 axis. World J Gastrointest Oncol. (2021) 13:2114–28. doi: 10.4251/wjgo.v13.i12.2114. PMID: 35070046 PMC8713308

[181] Sehm T Rauh M Wiendieck K Buchfelder M Eyüpoglu IY Savaskan NE . Temozolomide toxicity operates in a xCT/SLC7a11 dependent manner and is fostered by ferroptosis. Oncotarget. (2016) 7:74630–47. doi: 10.18632/oncotarget.11858. PMID: 27612422 PMC5342691

[182] Yeon Kim S Tang M Lu T Chih SY Li W . Ferroptosis in glioma therapy: advancements in sensitizing strategies and the complex tumor-promoting roles. Brain Res. (2024) 1840:149045. doi: 10.1016/j.brainres.2024.149045. PMID: 38821335 PMC11323215

[183] Zhao L Zhou X Xie F Zhang L Yan H Huang J . Ferroptosis in cancer and cancer immunotherapy. Cancer Commun (Lond). (2022) 42:88–116. doi: 10.1002/cac2.12250. PMID: 35133083 PMC8822596

[184] Abdel Baki SG Schwab B Haber M Fenton AA Bergold PJ . Minocycline synergizes with N-acetylcysteine and improves cognition and memory following traumatic brain injury in rats. PloS One. (2010) 5:e12490. doi: 10.1371/journal.pone.0012490. PMID: 20824218 PMC2930858

[185] Liu T Liu W Zhang M Yu W Gao F Li C . Ferrous-supply-regeneration nanoengineering for cancer-cell-specific ferroptosis in combination with imaging-guided photodynamic therapy. ACS Nano. (2018) 12:12181–92. doi: 10.1021/acsnano.8b05860. PMID: 30458111

[186] Zhang Y Fu X Jia J Wikerholmen T Xi K Kong Y . Glioblastoma therapy using codelivery of cisplatin and glutathione peroxidase targeting siRNA from iron oxide nanoparticles. ACS Appl Mater Interfaces. (2020) 12:43408–21. doi: 10.1021/acsami.0c12042. PMID: 32885649

[187] Nie Q Chen W Zhang T Ye S Ren Z Zhang P . Iron oxide nanoparticles induce ferroptosis via the autophagic pathway by synergistic bundling with paclitaxel. Mol Med Rep. (2023) 28(4):198. doi: 10.3892/mmr.2023.13085. PMID: 37681444 PMC10510030

[188] Fan R Chen C Mu M Chuan D Liu H Hou H . Engineering MMP-2 activated nanoparticles carrying B7-H3 bispecific antibodies for ferroptosis-enhanced glioblastoma immunotherapy. ACS Nano. (2023) 17:9126–39. doi: 10.1021/acsnano.2c12217. PMID: 37097811

[189] Shi W Yuan S Cheng G Zhang H Liu KJ Ji X . Blood brain barrier-targeted lipid nanoparticles improved the neuroprotection of Ferrostatin-1 against cerebral ischemic damage in an experimental stroke model. Exp Neurol. (2024) 379:114849. doi: 10.1016/j.expneurol.2024.114849. PMID: 38857748

[190] Monge-Fuentes V Biolchi Mayer A Lima MR Geraldes LR Zanotto LN Moreira KG . Dopamine-loaded nanoparticle systems circumvent the blood-brain barrier restoring motor function in mouse model for Parkinson's disease. Sci Rep. (2021) 11:15185. doi: 10.1038/s41598-021-94175-8. PMID: 34312413 PMC8313547

[191] Qi M Cheng Y Liu K Cai J Liu T Wu X . Recombinant human heavy chain ferritin nanoparticles serve as ROS scavengers for the treatment of ischemic stroke. Int J Nanomedicine. (2024) 19:2285–99. doi: 10.2147/ijn.s449606. PMID: 38482520 PMC10932756

[192] Jing L Xiao W Hu Z Liu X Yuan M . A systematic review of nanoparticle-mediated ferroptosis in glioma therapy. Int J Nanomedicine. (2025) 20:5779–97. doi: 10.2147/ijn.s523008. PMID: 40351706 PMC12065465

[193] Fernández-Mendívil C Luengo E Trigo-Alonso P García-Magro N Negredo P López MG . Protective role of microglial HO-1 blockade in aging: implication of iron metabolism. Redox Biol. (2021) 38:101789. doi: 10.1016/j.redox.2020.101789, PMID: 33212416 PMC7680814

[194] Wang Y Wu S Li Q Sun H Wang H . Pharmacological inhibition of ferroptosis as a therapeutic target for neurodegenerative diseases and strokes. Adv Sci (Weinh). (2023) 10:e2300325. doi: 10.1002/advs.202300325. PMID: 37341302 PMC10460905

[195] Tuo QZ Zhang ST Lei P . Mechanisms of neuronal cell death in ischemic stroke and their therapeutic implications. Med Res Rev. (2022) 42:259–305. doi: 10.1002/med.21817. PMID: 33957000

[196] Kakhlon O Manning H Breuer W Melamed-Book N Lu C Cortopassi G . Cell functions impaired by frataxin deficiency are restored by drug-mediated iron relocation. Blood. (2008) 112:5219–27. doi: 10.1182/blood-2008-06-161919. PMID: 18796625

[197] Mahoney-Sanchez L Bouchaoui H Boussaad I Jonneaux A Timmerman K Berdeaux O . Alpha synuclein determines ferroptosis sensitivity in dopaminergic neurons via modulation of ether-phospholipid membrane composition. Cell Rep. (2022) 40:111231. doi: 10.1016/j.celrep.2022.111231. PMID: 36001957

[198] Sauerbeck A Schonberg DL Laws JL McTigue DM . Systemic iron chelation results in limited functional and histological recovery after traumatic spinal cord injury in rats. Exp Neurol. (2013) 248:53–61. doi: 10.1016/j.expneurol.2013.05.011. PMID: 23712107 PMC5503200

[199] Jia H Liu X Cao Y Niu H Lan Z Li R . Deferoxamine ameliorates neurological dysfunction by inhibiting ferroptosis and neuroinflammation after traumatic brain injury. Brain Res. (2023) 1812:148383. doi: 10.1016/j.brainres.2023.148383. PMID: 37149247

[200] Imai T Tsuji S Matsubara H Ohba T Sugiyama T Nakamura S . Deferasirox, a trivalent iron chelator, ameliorates neuronal damage in hemorrhagic stroke models. Naunyn Schmiedebergs Arch Pharmacol. (2021) 394:73–84. doi: 10.1007/s00210-020-01963-6. PMID: 32808069

[201] Cheng Y Qu W Li J Jia B Song Y Wang L . Ferristatin II, an iron uptake inhibitor, exerts neuroprotection against traumatic brain injury via suppressing ferroptosis. ACS Chem Neurosci. (2022) 13:664–75. doi: 10.1021/acschemneuro.1c00819. PMID: 35143157

[202] Chen C Li M Lin L Chen S Chen Y Hong L . Clinical effects and safety of edaravone in treatment of acute ischaemic stroke: a meta-analysis of randomized controlled trials. J Clin Pharm Ther. (2021) 46:907–17. doi: 10.1111/jcpt.13392. PMID: 33638896 PMC8359409

[203] Soares P Silva C Chavarria D Silva FSG Oliveira PJ Borges F . Drug discovery and amyotrophic lateral sclerosis: emerging challenges and therapeutic opportunities. Ageing Res Rev. (2023) 83:101790. doi: 10.1016/j.arr.2022.101790. PMID: 36402404

[204] Yang J Liu M Zhou J Zhang S Lin S Zhao H . Edaravone for acute intracerebral haemorrhage. Cochrane Database Syst Rev. (2011) 2011(2):Cd007755. doi: 10.1002/14651858.cd007755.pub2. PMID: 21328300 PMC12665456

[205] Zhou Z Luo H Yu H Liu Z Zhong J Xiong J . Ferrostatin-1 facilitated neurological functional rehabilitation of spinal cord injury mice by inhibiting ferroptosis. Eur J Med Res. (2023) 28:336. doi: 10.1186/s40001-023-01264-7. PMID: 37697399 PMC10494332

[206] Li Q Han X Lan X Gao Y Wan J Durham F . Inhibition of neuronal ferroptosis protects hemorrhagic brain. JCI Insight. (2017) 2:e90777. doi: 10.1172/jci.insight.90777. PMID: 28405617 PMC5374066

[207] Guo M Zhu Y Shi Y Meng X Dong X Zhang H . Inhibition of ferroptosis promotes retina ganglion cell survival in experimental optic neuropathies. Redox Biol. (2022) 58:102541. doi: 10.1016/j.redox.2022.102541. PMID: 36413918 PMC9679710

[208] Skouta R Dixon SJ Wang J Dunn DE Orman M Shimada K . Ferrostatins inhibit oxidative lipid damage and cell death in diverse disease models. J Am Chem Soc. (2014) 136:4551–6. doi: 10.1021/ja411006a. PMID: 24592866 PMC3985476

[209] Xu Y Li K Zhao Y Zhou L Liu Y Zhao J . Role of ferroptosis in stroke. Cell Mol Neurobiol. (2023) 43:205–22. doi: 10.1007/s10571-022-01196-6. PMID: 35102454 PMC11415219

[210] Takeuchi S Wada K Nagatani K Otani N Osada H Nawashiro H . Sulfasalazine and temozolomide with radiation therapy for newly diagnosed glioblastoma. Neurol India. (2014) 62:42–7. doi: 10.4103/0028-3886.128280. PMID: 24608453

[211] Codenotti S Poli M Asperti M Zizioli D Marampon F Fanzani A . Cell growth potential drives ferroptosis susceptibility in rhabdomyosarcoma and myoblast cell lines. J Cancer Res Clin Oncol. (2018) 144:1717–30. doi: 10.1007/s00432-018-2699-0. PMID: 29971532 PMC11813460

[212] Fu C Wu Y Liu S Luo C Lu Y Liu M . Rehmannioside A improves cognitive impairment and alleviates ferroptosis via activating PI3K/AKT/Nrf2 and SLC7A11/GPX4 signaling pathway after ischemia. J Ethnopharmacol. (2022) 289:115021. doi: 10.1016/j.jep.2022.115021. PMID: 35091012

[213] Xiao S Wang C Yang Q Xu H Lu J Xu K . Rea regulates microglial polarization and attenuates neuronal apoptosis via inhibition of the NF-κB and MAPK signalings for spinal cord injury repair. J Cell Mol Med. (2021) 25:1371–82. doi: 10.1111/jcmm.16220. PMID: 33369103 PMC7875927

[214] Fan W Zhou J . Icariside II suppresses ferroptosis to protect against MPP(+)-induced Parkinson's disease through Keap1/Nrf2/GPX4 signaling. Chin J Physiol. (2023) 66:437–45. doi: 10.4103/cjop.cjop-d-23-00107. PMID: 38149556

[215] Li Y Meng F . Effects of icariside II on brain tissue oxidative stress and Nrf2/HO-1 expression in rats with cerebral ischemia-reperfusion injury1. Acta Cir Bras. (2019) 34:e201900208. doi: 10.1590/s0102-8650201900208. PMID: 30843941 PMC6585918

[216] Zhou J Deng Y Li F Yin C Shi J Gong Q . Icariside II attenuates lipopolysaccharide-induced neuroinflammation through inhibiting TLR4/MyD88/NF-κB pathway in rats. BioMed Pharmacother. (2019) 111:315–24. doi: 10.1016/j.biopha.2018.10.201. PMID: 30590319

[217] Zhang L Lang F Feng J Wang J . Review of the therapeutic potential of Forsythiae Fructus on the central nervous system: active ingredients and mechanisms of action. J Ethnopharmacol. (2024) 319:117275. doi: 10.1016/j.jep.2023.117275. PMID: 37797873

[218] Xie R Zhao W Lowe S Bentley R Hu G Mei H . Quercetin alleviates kainic acid-induced seizure by inhibiting the Nrf2-mediated ferroptosis pathway. Free Radic Biol Med. (2022) 191:212–26. doi: 10.1016/j.freeradbiomed.2022.09.001. PMID: 36087883

[219] Li L Jiang W Yu B Liang H Mao S Hu X . Quercetin improves cerebral ischemia/reperfusion injury by promoting microglia/macrophages M2 polarization via regulating PI3K/Akt/NF-κB signaling pathway. BioMed Pharmacother. (2023) 168:115653. doi: 10.1016/j.biopha.2023.115653. PMID: 37812891

[220] Lin ZH Liu Y Xue NJ Zheng R Yan YQ Wang ZX . Quercetin protects against MPP(+)/MPTP-induced dopaminergic neuron death in Parkinson's disease by inhibiting ferroptosis. Oxid Med Cell Longev. (2022) 2022:7769355. doi: 10.1155/2022/7769355. PMID: 36105483 PMC9467739

[221] Xiao L Dai Z Tang W Liu C Tang B . Astragaloside IV alleviates cerebral ischemia-reperfusion injury through NLRP3 inflammasome-mediated pyroptosis inhibition via activating Nrf2. Oxid Med Cell Longev. (2021) 2021:9925561. doi: 10.1155/2021/9925561. PMID: 35003524 PMC8739174

[222] Wang L Liu C Wang L Tang B . Astragaloside IV mitigates cerebral ischaemia-reperfusion injury via inhibition of P62/Keap1/Nrf2 pathway-mediated ferroptosis. Eur J Pharmacol. (2023) 944:175516. doi: 10.1016/j.ejphar.2023.175516. PMID: 36758783

[223] Shi L Chen H Chen K Zhong C Song C Huang Y . The DRD2 antagonist haloperidol mediates autophagy-induced ferroptosis to increase temozolomide sensitivity by promoting endoplasmic reticulum stress in glioblastoma. Clin Cancer Res. (2023) 29:3172–88. doi: 10.1158/1078-0432.ccr-22-3971. PMID: 37249604

